# Integrative bioinformatics, network toxicology, and molecular docking elucidate molecular mechanisms of ATBC-induced sarcoma progression with experimental validation

**DOI:** 10.1186/s40360-026-01141-z

**Published:** 2026-04-24

**Authors:** Yue Wang, Xuan Lin, Yihuang Chen, Yuanqun Zhang, Qiang Zhang, Miao Xu, Xiaoning Lin, Xin Wu, Shifu Peng, Jianlin Shen

**Affiliations:** 1 Central Laboratory, School of Basic Medicine, Affiliated Hospital of Putian University, Putian University, Putian, Fujian 351100 China; 2 Key Laboratory of Translational Tumor Medicine in Fujian Province, Putian University, Putian, Fujian 351100 China; 3 College of Environmental and Biological Engineering, Putian University, Putian, Fujian Province 351100 China; 4 School of Basic Medicine, Fujian Medical University, Fuzhou, Fujian 350100 China; 5 Department of Orthopedics, Affiliated Hospital of Putian University, Putian, Fujian 351100 China; 6 Department of Environment and Health, Jiangsu Center for Disease Control and Prevention, Nanjing, Jiangsu 210009 China; 7 Central Laboratory, Affiliated Hospital of Putian University, Putian, Fujian Province 351100 China

**Keywords:** Sarcoma progression, ATBC, Mechanism, Network toxicology, Molecular docking

## Abstract

**Background:**

Acetyl tributyl citrate (ATBC), a widely used plasticizer, has raised health concerns due to its potential environmental persistence and human exposure, but its toxicological effects on sarcoma remain unclear.

**Methods:**

We employed an integrated approach combining network toxicology, molecular docking, survival analysis, and experimental validation to systematically investigate ATBC’s impact on sarcoma.

**Results:**

Multi-database screening identified 102 overlapping targets. Protein–protein interaction analysis revealed six hub genes: TLR4, ESR1, PPARG, SIRT1, NFKB1, and PTGS2. Functional enrichment analysis indicated significant involvement in cancer-related pathways, including PI3K–Akt, HIF‑1, and AGE–RAGE signaling. Molecular docking predicted potential interactions between ATBC and these targets, with binding energies ranging from -1.15 to -2.99 kcal/mol. Survival analysis associated high PPARG expression with poor prognosis, while high TLR4 and ESR1 expression correlated with better survival. In vitro experiments showed ATBC promoted proliferation and migration of sarcoma cells. qPCR results confirmed ATBC downregulated TLR4 and ESR1 and upregulated PPARG, aligning with clinical prognostic trends.

**Conclusions:**

These findings suggest ATBC may exert tumor-promoting effects by modulating core targets and pathways, highlighting its potential role in sarcoma progression and the importance of environmental health risk assessment.

**Supplementary Information:**

The online version contains supplementary material available at 10.1186/s40360-026-01141-z.

## Introduction

Acetyl tributyl citrate (ATBC) is an organic ester produced by esterifying citric acid. Owing to its high chemical stability and good material compatibility, it serves as a common plasticizer within polyvinyl chloride and related polymeric substances [1]. Currently, ATBC is considered a low-toxicity plasticizer, with adverse effects typically occurring only under very high exposure conditions. The European Food Safety Authority established a no-observed-adverse-effect level of 100 mg/kg body weight per day and derived a tolerable daily intake of 1.0 mg/kg body weight for ATBC [2, 3]. As a result, ATBC is authorized by European Union legislation for unrestricted application in food-contact plastics. Additionally, The U.S. FDA approves this substance as a pharmaceutical excipient, functioning both as a drug carrier and a coating for tablets and capsules [4, 5].

However, as ATBC finds broader application, its high migration potential has drawn increasing attention. The migration of ATBC from plastic products, including food packaging and medical devices, is attributed to the absence of covalent integration with the polymer matrix, thereby facilitating its mobilization into the surrounding environment [6]. As a result, humans can be exposed to ATBC through numerous routes, such as consuming tainted food and water, inhaling polluted air and dust, skin contact, using certain medical devices, and taking medications with ATBC coatings. An FDA report indicates that daily intake of the substance from some pills or capsules can reach 20.2 mg [7]. Alves et al. reported that ATBC is eliminated via urine within about two days, whereas it can be deposited in nail tissue for periods as long as six months [8]. These findings suggest that ATBC is ubiquitous in the human environment. Its relatively high solubility enhances bioavailability, facilitating entry into the body and contributing to its persistence and bioaccumulation in humans. Given the expanding applications of ATBC and its marked migration-leaching behavior, there is an urgent need to develop new strategies for comprehensive health risk evaluations and research to elucidate its underlying toxicological mechanisms.

Recent studies have raised widespread concern regarding the potential health risks posed by ATBC to humans. Multiple toxicological investigations have revealed its endocrine-disrupting effects, including anti-estrogenic and anti-androgenic activities, disruption of thyroid hormone homeostasis, and adverse impacts on glucose and lipid metabolism. These effects may be mediated through mechanisms such as disruption of metabolic pathways, triggering of inflammatory responses, or compromise of cellular homeostasis [9–12]. Furthermore, animal experiments indicate that when mothers are exposed to ATBC during pregnancy, it can cause transgenerational disturbances in the glucose metabolism of their offspring [13], underscoring the importance of evaluating the association between ATBC exposure and long-term health risks in humans. While current research has primarily focused on metabolic disorders resulting from ATBC-induced endocrine disruption, its potential role in promoting tumor progression remains poorly understood and warrants further investigation.

Sarcoma is a group of heterogeneous malignant neoplasms derived from mesenchymal tissue, comprising more than 70 subtypes that are broadly classified into soft tissue sarcoma (STS) and osteosarcoma. Although rare, sarcomas exhibit diverse pathological types and are associated with a high postoperative recurrence rate [14]. Advanced sarcoma carries a dismal prognosis, evidenced by a five-year overall survival rate of merely 30–40% [15]. Advances in sequencing technologies have prompted recent studies to focus on gene mutations in sarcoma patients, which play important roles in tumor initiation and progression [16–18]. Nevertheless, the etiology of most sarcomas remains unclear, and their increasing annual incidence poses a significant medical challenge [19]. Studies have also suggested that genetic factors, dietary habits, and environmental exposures may play a role in sarcoma [20–22]. The rising incidence of sarcoma has been partly attributed to environmental factors, with long-term exposure to chemicals such as plasticizers suspected of having a promoting effect [23]. Therefore, in-depth investigation into the molecular mechanisms by which the plasticizer ATBC may promote sarcoma development significantly to the formulation of strategies for sarcoma prevention.

Integrating network toxicology with molecular docking provides an effective approach for uncovering toxicological mechanisms. Originally developed in 2007, network pharmacology has established itself as an important methodology based on systems biology and bioinformatics to decipher molecular mechanisms of drug action [24]. It focuses on complex interaction networks among drugs, pathways, targets, and diseases, and is particularly suitable for systematic interpretation of multi-target drug mechanisms in areas such as psychiatry, oncology, and anti-infective therapy [25]. Building on this foundation, network toxicology extends the principles of network pharmacology and network biology by incorporating bioinformatics, big data analysis, genomics, proteomics, and metabolomics to deeply investigate compound toxicity and biological interactions [26]. This method constructs association networks among compounds, toxic effects, and targets to visualize and systematically analyze multi-component, multi-target toxicity, thereby predicting disease mechanisms from toxicant exposure [27]. Rooted in computer-aided drug design, molecular docking is a pivotal computational method for evaluating ligand-target interactions by computing their binding energy, where lower values indicate more stable binding conformations and more favorable interactions [28].

**Fig. 1:**
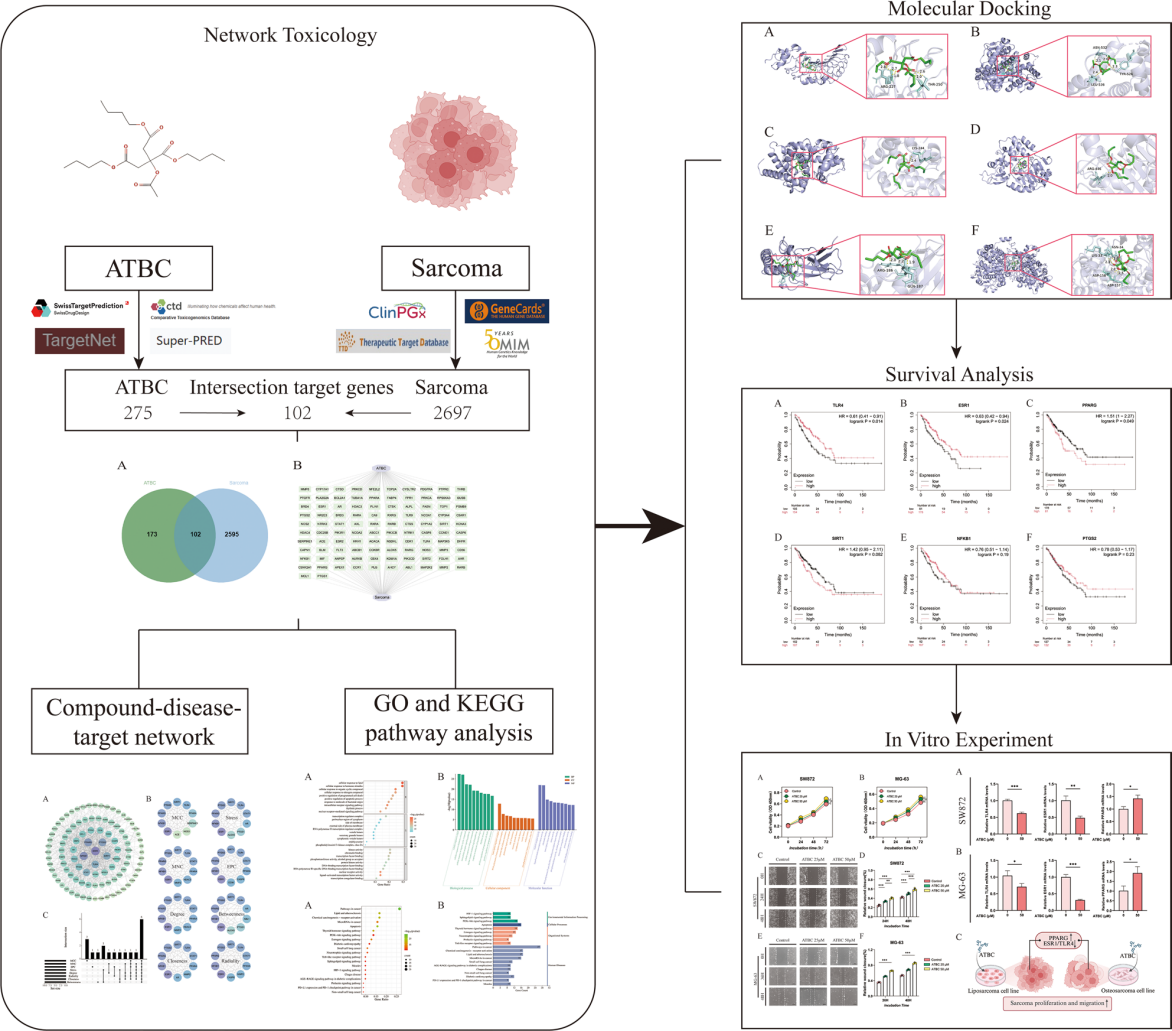
Flowchart of the mechanistic analysis of ATBC-promoted sarcoma

This study employed an integrated approach combining network toxicology, molecular docking, survival analysis, and in vitro experiments to assess the impact of ATBC on sarcoma. We screened potential carcinogenic targets of ATBC across multiple databases and applied topological algorithms to identify core targets. Based on these findings, we developed a network-based predictive model and employed network toxicology methods to systematically identify and analyze toxic pathways associated with potential ATBC-induced sarcoma risk. This approach aims to determine the carcinogenic potential of ATBC and to elucidate the molecular mechanisms underlying its promotion of sarcoma development and progression. To evaluate the influence of the core target genes on overall survival in sarcoma patients, we constructed prognostic landscapes. Subsequent in vitro experimental validation demonstrated that ATBC exposure promotes sarcoma cell proliferation and migration (Fig. 1). Through this comprehensive investigation, we aim to uncover the molecular basis of potential ATBC-associated carcinogenic effects and identify potential targets for mitigating its adverse effects. Moreover, the research provides a critical reference for the toxicity evaluation of frequently encountered chemicals and lays the groundwork for diagnosing related exposure-associated diseases.

## Materials and methods

### Toxicity analysis of ATBC

We evaluated the potential toxicity of the target compounds using the toxicity prediction tool ProTox (available at https://tox.charite.de/protox3/). Basic toxicological properties of the compounds of interest (ATBC) were obtained from the ProTox platform.

### Acquisition of ATBC targets

The initial screening for potential target proteins of ATBC involved obtaining its standard chemical structure and SMILES identifier from the PubChem database. Using “acetyl tributyl citrate” as the search term, potential targets were screened from the Swiss Target Prediction, Super-PRED, TargetNet, and Comparative Toxicogenomics Database (CTD) platforms, with species limited to “Homo sapiens”. Following structural verification and consistency checks of the retrieved targets, the predicted targets were standardized via the UniProt database, resulting in a merged and deduplicated set of potential ATBC targets (Table 1).

**Table 1:** Target source information for ATBC and Sarcoma

Source	Website	Number of targets	Type	Filtration Options
SwissTargetPrediction	http://swisstargetprediction.ch/	13	ATBC	Probability > 0
TargetNet	http://targetnet.scbdd.com/	138	ATBC	Probability > 0
SuperPred	https://prediction.charite.de/subpages/target_prediction.php	119	ATBC	Probability ≥ 50%
CTD	https://www.ctdbase.org/	47	ATBC	-
GeneCards	https://www.genecards.org/	2639	Sarcoma	Relevance score ≥ median
OMIM	https://www.omim.org/	73	Sarcoma	-
PharmGKB	https://www.clinpgx.org/	36	Sarcoma	-
TTD	https://db.idrblab.net/ttd/	31	Sarcoma	-

Note: SwissTargetPrediction: Swiss Drug Target Prediction Database; TargetNet: Target Netting; SuperPred: Prediction of Drug Targets and Off-targets; CTD: Comparative Toxicogenomics Database; GeneCards: The Human Gene Database‌; OMIM: Online Mendelian Inheritance in Man; PharmGKB: Pharmacogenetics and Pharmacogenomics Knowledge Base; TTD: Therapeutic Target Database

### Acquisition of sarcoma-related targets

Potential therapeutic targets for sarcoma were compiled through a systematic search of the GeneCards, OMIM, PharmGKB, and Therapeutic Target Database (TTD) platforms, using “sarcoma” as the primary search term. To ensure high relevance between the selected genes and sarcoma, genes obtained from GeneCards were filtered based on their “Relevance score”, with the median score set as the threshold. Only genes scoring above the median were retained. Data from all four databases were integrated, duplicates were removed, and a non-redundant set of sarcoma-related targets was established (Table 1). Subsequently, a Venn diagram analysis was utilized to determine the intersection between the predicted ATBC targets and the sarcoma-related target set. These common targets were recognized as the potential therapeutic targets through which ATBC may mediate its underlying mechanism in sarcoma.

### Protein-protein interaction (PPI) network construction and core target screening

The overlapping target genes were imported into the STRING database using the species parameter “Homo sapiens” and a medium confidence interaction score threshold of 0.400 as the minimum required criterion. A protein-protein interaction (PPI) network was then constructed and its topological parameters were calculated using the Network Analyzer module within Cytoscape software (version 3.10.1). A multi-algorithm integration strategy was implemented via the CytoHubba plugin, incorporating eight topological analysis methods—MCC, MNC, EPC, Degree, Closeness, Betweenness, Radiality, and Stress—to screen the top 10 key genes ranked by each algorithm. The results from all eight algorithms were further integrated, leading to the identification of six core regulatory targets with multi-algorithm consensus.

### Functional enrichment and pathway analysis of targets

Bioinformatic analysis of the intersecting targets was conducted using the Metascape database, which involved performing Gene Ontology (GO) enrichment analysis, as well as Kyoto Encyclopedia of Genes and Genomes (KEGG) pathway analysis to elucidate the principal biological functions and identify the key toxicity-related pathways associated with these targets.

### Molecular docking

Molecular docking simulations were utilized to forecast both the binding energy and three-dimensional binding conformation of ATBC with the core proteins discovered in this investigation, thereby facilitating further analysis of their intermolecular interaction mechanisms. Three-dimensional structural data for the principal targets were acquired from the RCSB Protein Data Bank (PDB), with subsequent refinement using PyMOL software to remove hydration molecules and endogenous ligands. The prepared protein structures underwent molecular optimization in AutoDockTools 1.5.7 through hydrogen addition, charge calculation, and non-polar hydrogen unification. The AutoGrid4 and AutoDock4 modules integrated in AutoDockTools facilitated successful docking of the ligand (ATBC) with respective receptor structures, enabling evaluation of binding stability between the toxicant and its protein target, while the outcomes of the molecular docking were graphically represented through PyMOL software.

### Survival analysis

Using the KM Plotter pan-cancer RNA-seq module (https://kmplot.com/analysis), we performed exploratory survival analysis on the TCGA-based sarcoma cohort (*n* = 259). Patients were dichotomized using the platform’s auto-selected cutoff, and overall survival was evaluated by log-rank test. Because this web-based analysis used the pooled sarcoma dataset available in the public platform, subtype-stratified and multivariate Cox analyses were not performed in the present study.

### Cell culture

SW872 and MG-63 cell lines (acquired from ATCC, Manassas, VA, United States) were maintained in high-glucose Dulbecco’s Modified Eagle Medium (DMEM; Gibco) containing 10% fetal bovine serum (FBS; Gibco) and 1% penicillin (Gibco). The cells were grown in a humidified incubator at 37°C with 5% CO₂.

### ATBC solution preparation

Acetyl tributyl citrate (ATBC, CAS No. 77–90–7, purity > 98%) was acquired from Sigma-Aldrich (St. Louis, MO, USA), while dimethyl sulfoxide (DMSO) was supplied by Solarbio (Beijing, China). To generate the stock solution, ATBC was solubilized in DMSO and subsequently subjected to dilution using cell culture medium to reach the necessary working concentrations, with the ultimate DMSO level consistently held under 0.1% (v/v) across all investigative protocols. The working concentrations of 25 and 50 µM were selected based on preliminary cytotoxicity assays and reference to previous in vitro toxicological studies of plasticizers [6].

### CCK-8 assay

SW872 and MG-63 cell lines were seeded into 96-well plates and exposed to ATBC at 0, 25, and 50 µM concentrations. Following treatment periods of 24, 48, and 72 h, 10 µL of CCK-8 reagent (HY-K0301; MCE) was added to each well, with subsequent incubation for 2 h at 37°C in a 5% CO₂ atmosphere. Optical density measurements were then taken at 450 nm wavelength using a multimode microplate reader (Tecan Spark, Switzerland).

### Wound healing assay

SW872 and MG-63 cells were plated in 6-well plates and cultured until reaching confluence, after which a standardized wound was introduced into the monolayer using a sterile pipette tip. Subsequently, the culture medium was exchanged for serum-free formulation supplemented with ATBC at 0, 25, or 50 µM. Wound images were acquired at 0, 36, and 48 h post-treatment using an inverted fluorescence microscope (Nikon Eclipse Ti2-U, Japan). The wound areas were quantified with ImageJ software, and healing rates were statistically analyzed to compare the effects of different treatments and time points.

### RT-qPCR assay

Total RNA was extracted from cells using TRIzol reagent. Reverse transcription was performed using the PrimeScript RT Premix (Takara, Dalian, China) to synthesize cDNA from the extracted RNA. The cDNA was then amplified using Hieff qPCR SYBR Green Master Mix (High Rox Plus) (Yeasen, Shanghai, China), and quantitative analysis was carried out on the ABI StepOne Plus Detection System (Thermo Fisher Scientific, Massachusetts, USA). For RT-qPCR validation, cells were treated with vehicle control or 50 µM ATBC. The 50 µM concentration was selected for this preliminary transcriptional analysis because it produced more evident phenotypic changes in the proliferation and migration assays.

Primers for TLR4, ESR1, PPARG, SIRT1, NFKB1, PTGS2, and GAPDH were designed using NCBI Primer-BLAST based on the corresponding human gene sequences, and primer specificity was checked before use. GAPDH was used as the internal reference gene for normalization, and relative mRNA expression was calculated using the 2^−ΔΔCt method. The processed normalized RT-qPCR results are provided in Supplementary Table S1. The primer sequences used in this study are listed in Table 2. Melt-curve analysis was performed after amplification to verify the specificity of each primer pair and confirm the presence of a single amplification product.

**Table 2:** Primer sequences used for RT-qPCR

Gene	Forward primer (5’-3’)	Reverse primer (5’-3’)
TLR4	TGGTGTCCCAGCACTTCATC	CTGTCCTCCCACTCCAGGTA
ESR1	GGTGCCCTACTACCTGGAGA	ACACTGCACAGTAGCGAGTC
PPARG	GCAATCAAAGTGGAGCCTGC	TCTCCGGAAGAAACCCTTGC
SIRT1	GGAGCAGATTAGTAGGCGGC	CCTCAGCGCCATGGAAAATG
NFKB1	AATGGGCTACACCGAAGCAA	TCTAGAGGTCCTTCCTGCCC
PTGS2	TTGCATTCTTTGCCCAGCAC	ACCGTAGATGCTCAGGGACT
GAPDH	GTCAAGGCTGAGAACGGGAA	AAATGAGCCCCAGCCTTCTC

### Statistical analysis

Experimental results are presented as mean ± SD (*n* = 3). For comparisons involving two groups, the t-test was applied, whereas one-way ANOVA was utilized when comparing multiple groups. A probability threshold of *P* < 0.05 was defined as statistical significance, and all statistical evaluations were executed with GraphPad Prism software (version 9.0).

## Results

### ATBC toxicity analysis

**Fig. 2:**
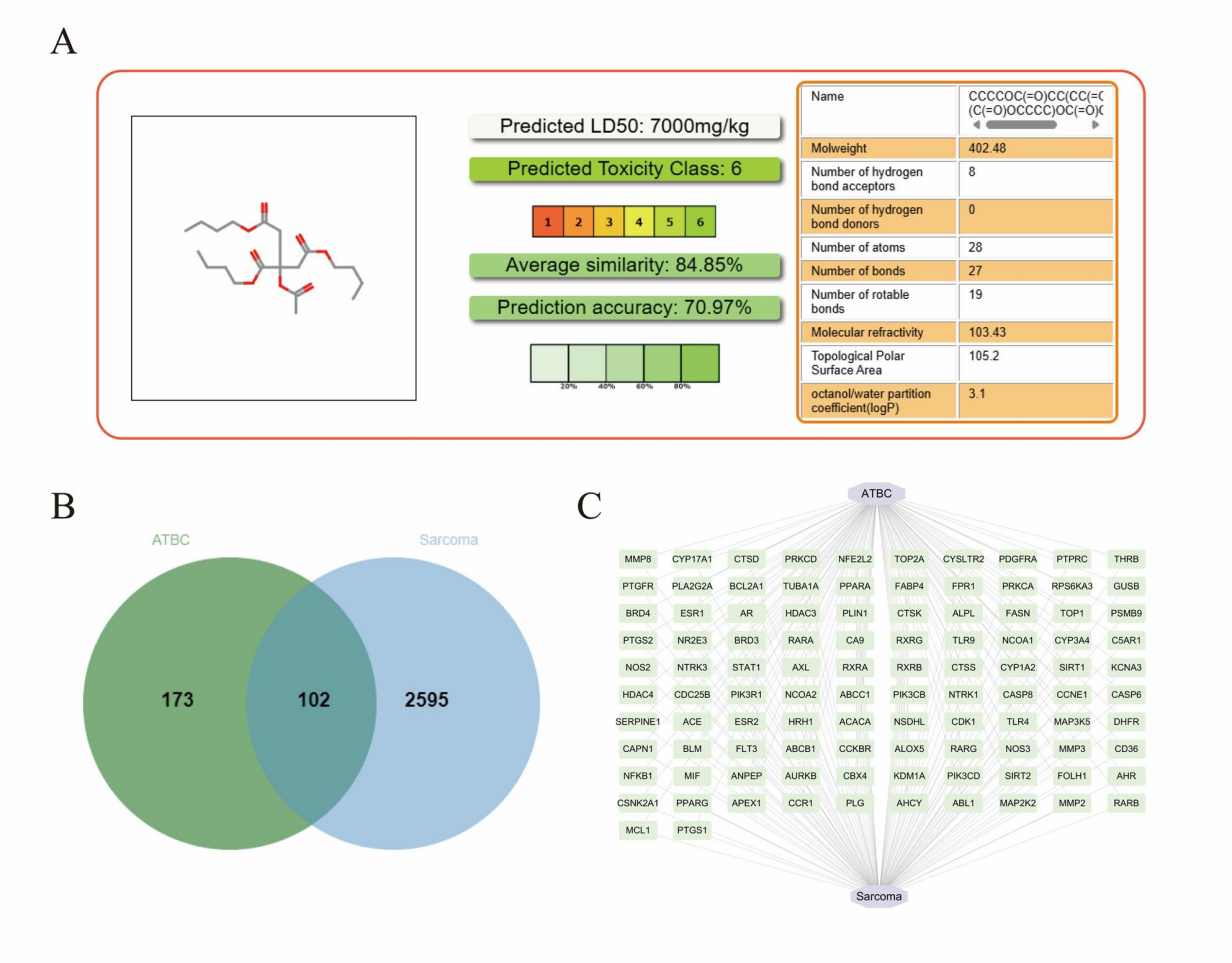
Identification of common targets between ATBC and sarcoma. (**A**) Structural representation and toxicological profiling of ATBC obtained from ProTox; (**B**) Venn diagram illustrating the shared targets between ATBC and sarcoma; (**C**) Network visualization generated through Cytoscape software depicting the 102 overlapping targets identified in the Venn analysis

According to the ProTox database prediction, the median lethal dose (LD50) of ATBC is 7000 mg/kg, corresponding to toxicity class 6. Furthermore, the database supplied critical molecular descriptors for this compound, comprising molecular weight, hydrogen bond acceptors and donors, atom and bond counts, rotatable bonds, molecular refractivity, and topological polar surface area (Fig. 2A). These parameters offer valuable insight into the physicochemical properties of ATBC and its potential carcinogenic effects.

### Identification of potential targets of ATBC in sarcoma

We identified potential target proteins of ATBC through searches in the Swiss Target Prediction, Super-PRED, TargetNet, and Comparative Toxicogenomics Database databases, yielding a total of 275 targets. Additionally, we retrieved 2,697 targets associated with osteosarcoma from the GeneCards, OMIM, PharmGKB, and Therapeutic Target Database databases. Integration and deduplication of these two datasets via a Venn diagram revealed 102 overlapping targets (Fig. 2B). These 102 overlapping targets were subsequently visualized using Cytoscape software (Fig. 2C).

### PPI networks of potential targets and identification of core genes

**Fig. 3:**
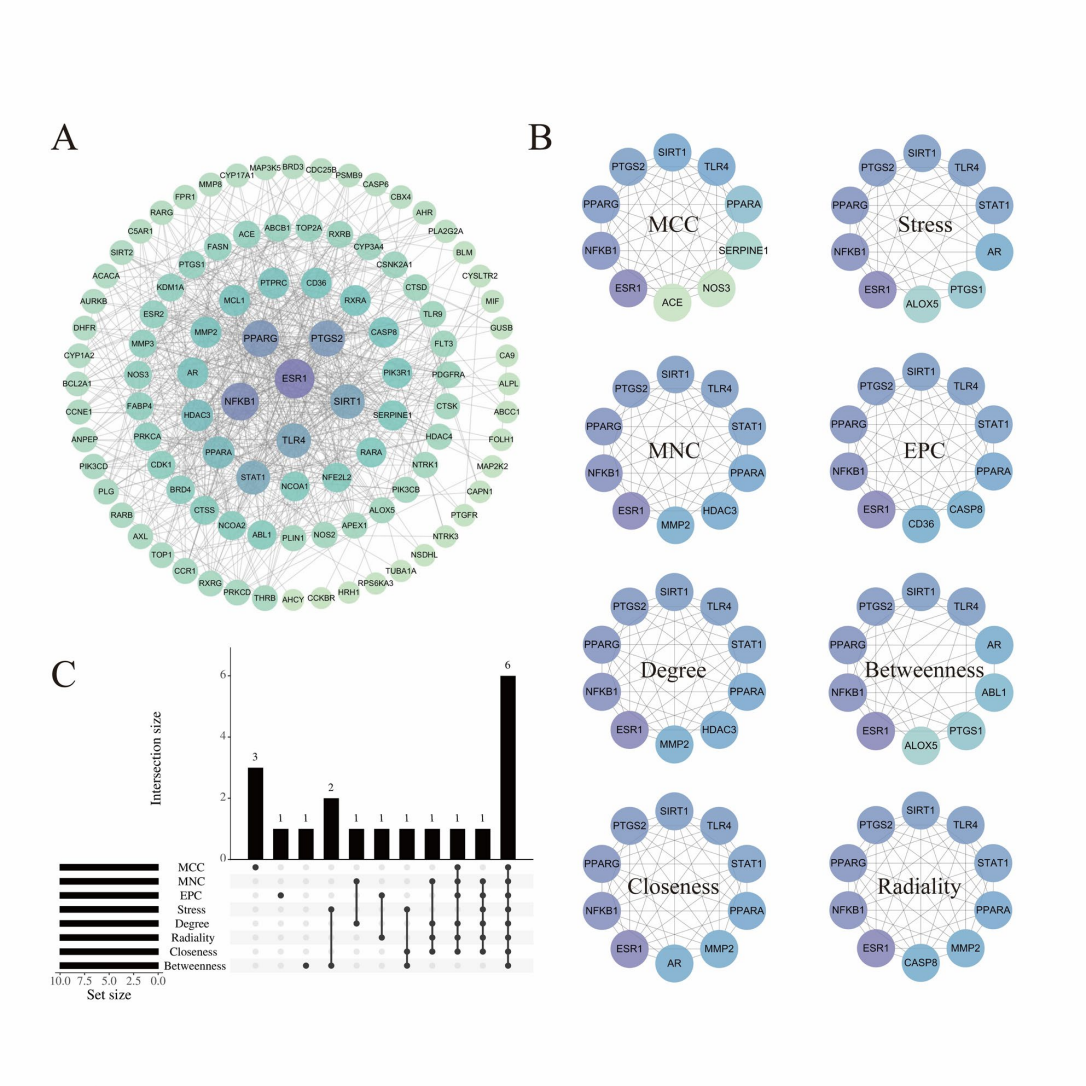
Collection of core gene targets of ATBC and sarcoma. (**A**) PPI network optimized with Cytoscape; (**B**) Top 10 genes screened by eight topological algorithms, with composite scores shown in a gradient colormap; (**C**) Intersection analysis of algorithmic results identified six targets with the highest pathological relevance (TLR4, ESR1, PPARG, SIRT1, NFKB1, PTGS2)

A protein-protein interaction (PPI) network was constructed using the STRING database, consisting of 100 nodes and 734 interaction edges. Cytoscape software was employed to analyze the topological properties of the network nodes and generate an optimized visualization of the PPI network (Fig. 3A). Node size and color were scaled according to their degree values, where larger dimensions and darker hues represent higher degrees and greater functional significance within the network. Using the CytoHubba plugin, we applied a fusion strategy incorporating eight topological algorithms (Stress, MNC, MCC, EPC, Betweenness, Radiality, Closeness, and Degree). The integrated results from these algorithms revealed several densely interconnected clusters (Fig. 3B). The intersection of results from all eight algorithms identified the six core targets: TLR4, ESR1, PPARG, SIRT1, NFKB1, and PTGS2 (Fig. 3C).

### GO and KEGG pathway enrichment analysis of potential targets

**Fig. 4:**
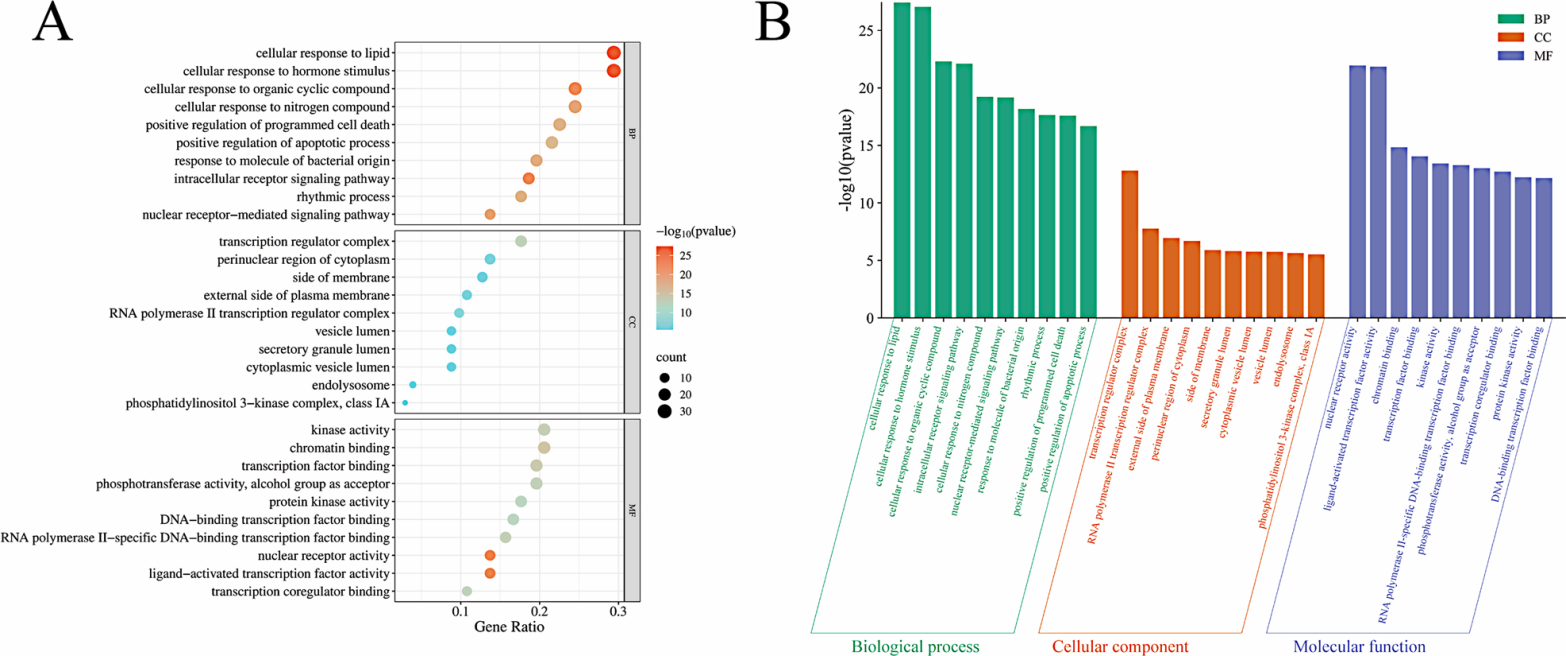
Gene Ontology enrichment assessment of 102 candidate targets highlighting the ten most significantly enriched terms. (**A**) The dimensions of the bubbles correspond to the quantity of genes linked to a particular pathway, whereas the saturation of the color reflects the statistical confidence of the enrichment evaluation. (**B**) From the GO enrichment analysis conducted on 102 potential targets, the bar graph illustrates the ten most substantially enriched terms spanning biological processes BP, CC, and MF. Evaluation of the enrichment outcomes relies on P-values, with reduced numerical magnitudes designating greater statistical importance. The vertical extent of each bar is directly proportional to the P-value, offering a graphical depiction of the enrichment intensity across diverse functional groupings

GO enrichment analysis was performed on 102 candidate targets through the Metascape platform under Homo sapiens species limitation, revealing 1173 statistically enriched GO terms distributed across biological process (BP, 970 terms), cellular component (CC, 55 terms), and molecular function (MF, 148 terms) categories. The terms were sorted by P-value, and the top 10 entries from BP, CC, and MF were selected for visualization in enrichment analysis plots (Fig. 4A&B). Enrichment results demonstrated principal involvement of core targets in BP including cellular response to lipid, cellular response to hormone stimulus, and cellular response to organic cyclic compound; CC were predominantly enriched in transcription regulator complex, RNA polymerase II transcription regulator complex, and external side of plasma membrane; and MF were characterized by nuclear receptor activity, ligand-activated transcription factor activity, and chromatin binding.

**Fig. 5:**
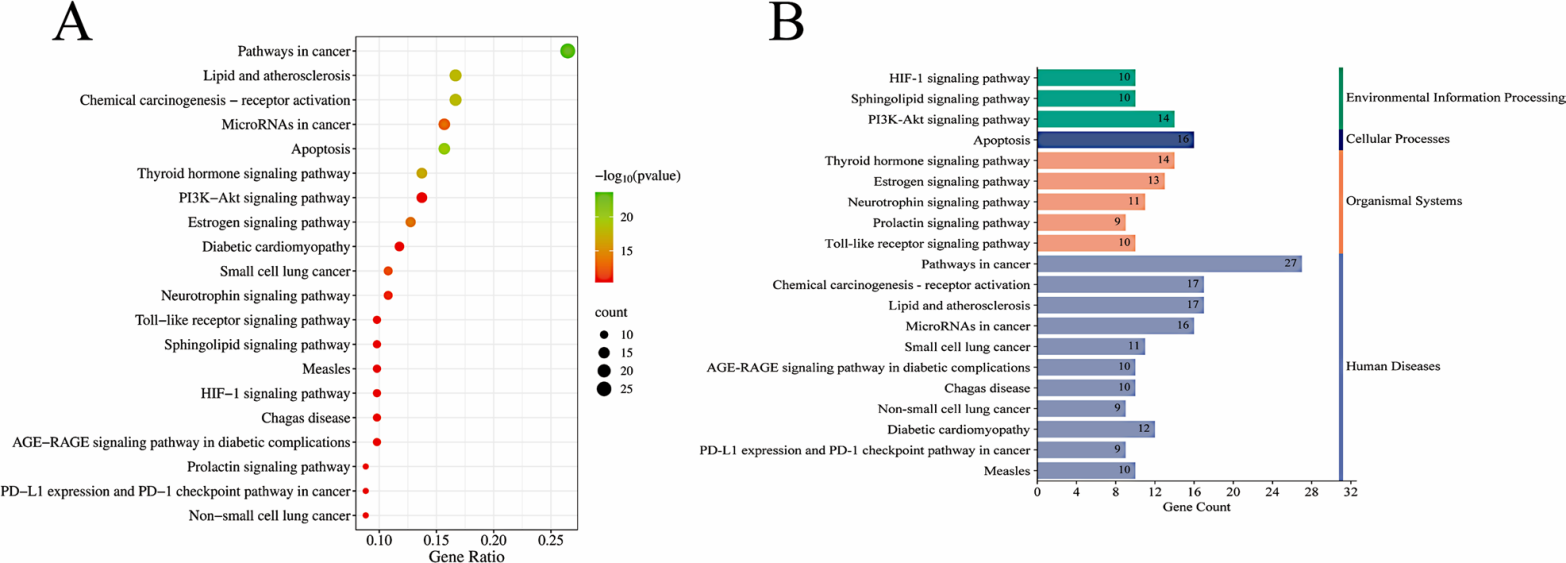
Kyoto Encyclopedia of Genes and Genomes enrichment examination of candidate targets (top 20). (**A**) The graphical representation illustrates the twenty most substantially enriched KEGG signaling pathways arranged in increasing P-value sequence. Individual bubbles correspond to distinct pathways, where dimensional area corresponds to enriched gene quantity and chromatic saturation denotes statistical confidence in enrichment. (**B**) The histogram presents pathway enrichment frequency and statistical confidence levels, where bar length exhibits positive correlation with gene quantity enriched in each pathway while concurrently demonstrating enrichment magnitude and statistical relevance. Longer bars indicate a greater number of enriched genes and stronger enrichment significance

Additionally, KEGG pathway enrichment analysis on the same targets using Metascape identified 155 significantly enriched signaling pathways, with the top 20 most significant pathways displayed in statistical bubble charts and categorical histograms ranked by ascending P-value (Fig. 5A&B). These pathways included Thyroid hormone, Estrogen, Neurotrophin, AGE-RAGE in diabetic complications, Prolactin, Toll-like receptor, HIF-1, Sphingolipid, and PI3K-Akt signaling pathways, indicating potential molecular mechanisms through which ATBC may affect sarcoma, along with associated biological processes and signaling pathways.

### Molecular docking of ATBC and sarcoma core target proteins

**Fig. 6:**
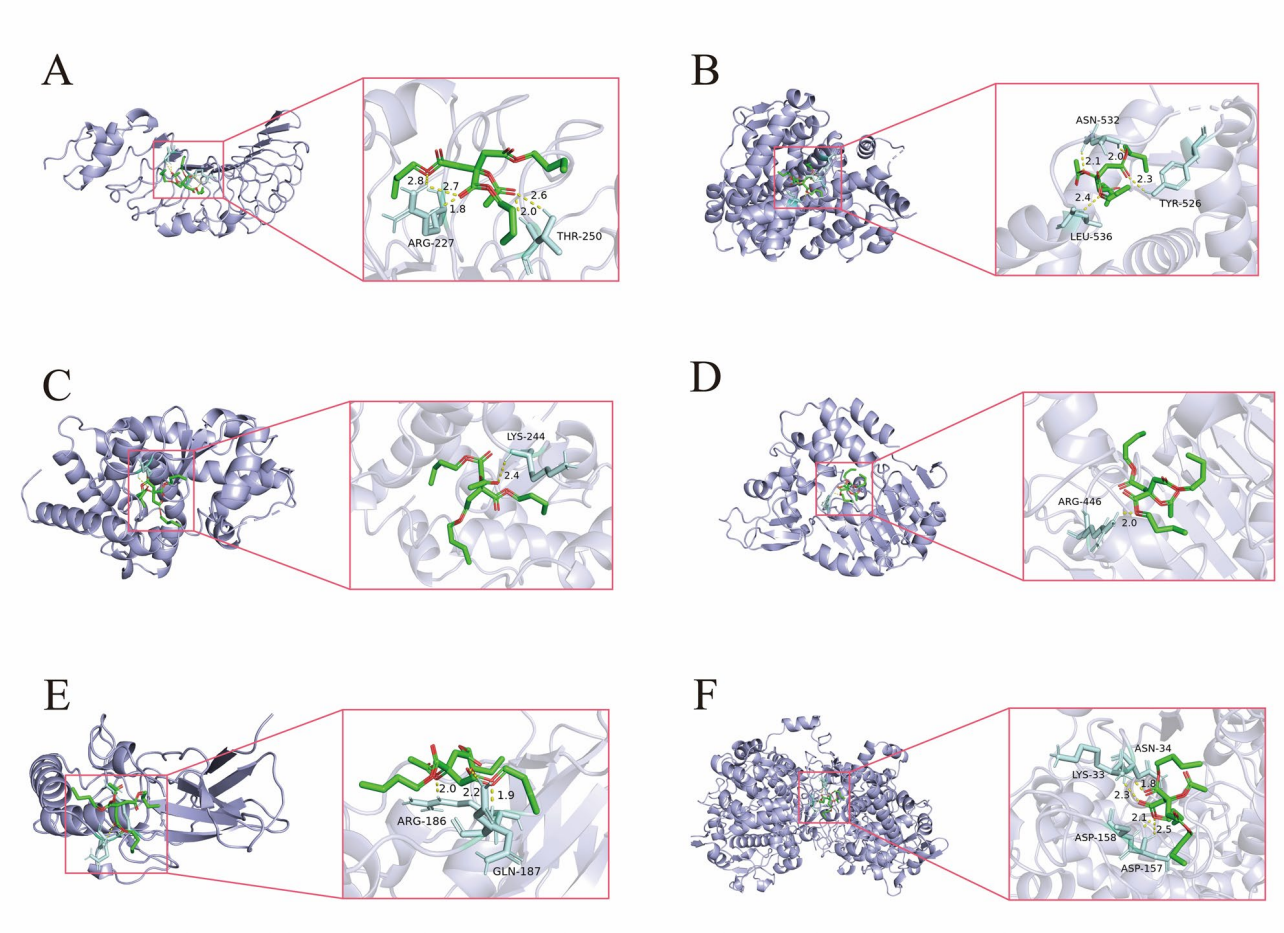
Molecular docking in each target protein with the ATBC. (**A**) ATBC and TLR4; (**B**) ATBC and ESR1; (**C**) ATBC and PPARG; (**D**) ATBC and SIRT1; (**E**) ATBC and NFKB1; (**F**) ATBC and PTGS2

Molecular docking simulations between ATBC and core target proteins were performed using AutoDock software. The results demonstrated spontaneous binding interactions between ATBC and six sarcoma-related core targets: TLR4 (PDB ID: 3ul9, -2.03 kcal/mol), ESR1 (PDB ID: 6psj, -2.78 kcal/mol), PPARG (PDB ID: 7e0a, -2.56 kcal/mol), SIRT1 (PDB ID: 4zzi, -2.99 kcal/mol), NFKB1 (PDB ID: 8tqd, -2.69 kcal/mol), and PTGS2 (PDB ID: 5f19, -1.15 kcal/mol). The binding energies ranged from -1.15 to-2.99 kcal/mol (Table 3), indicating thermodynamically favorable interactions. Although these values indicate relatively weak interactions, they suggest that ATBC may directly interact with these targets. The docking conformation with the lowest binding energy was visualized using PyMOL software (Fig. 6A − F), clearly illustrating the binding modes between ATBC and the core target proteins.

**Table 3:** Binding energies of ligands and receptors

ligand	receptor	Bind. Energy (Kcal/mol)
ATBC	TLR4	-2.03
ATBC	ESR1	-2.78
ATBC	PPARG	-2.56
ATBC	SIRT1	-2.99
ATBC	NFκB1	-2.69
ATBC	PTGS2	-1.15

### Impact of core targets expression on overall survival

**Fig. 7:**
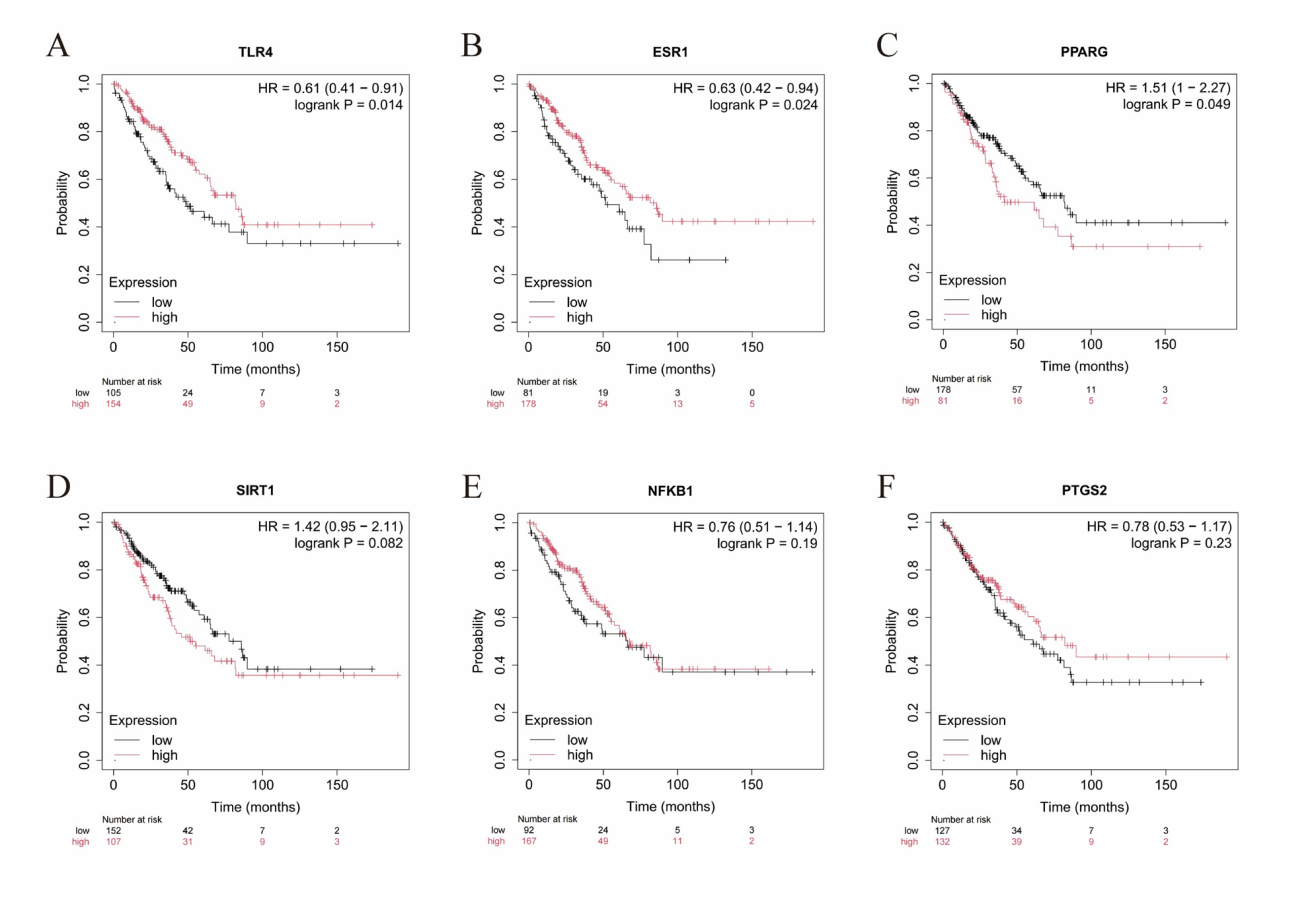
Analysis of overall survival for core targets in sarcoma. (**A**–**F**) Association between TLR4, ESR1, PPARG, SIRT1, NFKB1, and PTGS2 expression and patient prognosis

Exploratory Kaplan-Meier survival analysis was further conducted to evaluate the association between the expression levels of the six core genes and overall survival in the public sarcoma cohort. The survival curves demonstrated that subgroups with high expression of TLR4 or ESR1 exhibited significantly longer survival times (Fig. 7A&B). In contrast, high PPARG expression was significantly associated with poor survival and markedly reduced overall survival (Fig. 7C). Elevated expression of SIRT1, as well as reduced expression of NFKB1 or PTGS2, may also be associated with an increased mortality risk (Fig. 7D–F), although the Kaplan-Meier analysis did not reveal statistically significant associations between their expression levels and patient survival (*P* > 0.05). Because this analysis was based on a pooled public dataset and did not include multivariable adjustment or subtype-stratified analysis, these findings should be interpreted cautiously. This lack of significance may also be due to factors such as cohort heterogeneity or the relatively small sample size available in the online database.

### ATBC enhances sarcoma cell proliferation and migration in vitro

**Fig. 8:**
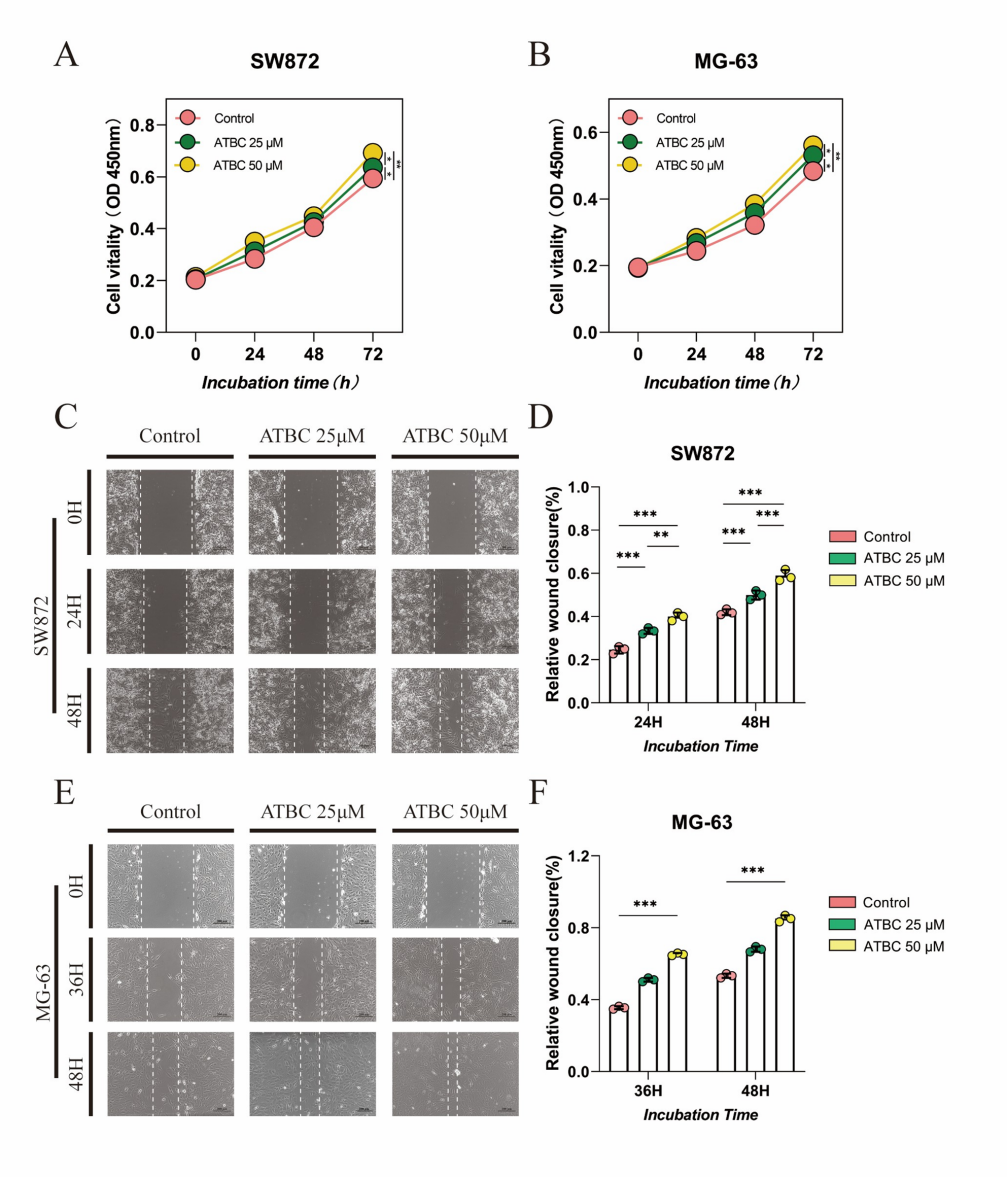
Results of the experimental study on the effects of ATBC on sarcoma cells. (**A**-**B**) CCK-8 assay assessing the effect of ATBC on SW872 and MG-63 cells proliferation; (**C**, **E**) Microscopic images of the scratch wound healing assay in SW872 and MG-63 cells; (**D**, **F**) Wound closure percentage of SW872 and MG-63 cells measured via the wound healing assay

To evaluate the direct impact of ATBC on sarcoma cells, two in vitro models—the human sarcoma SW872 and MG-63 cell lines—were employed to investigate its cancer-promoting effects across various concentrations. CCK-8 assays demonstrated that ATBC enhanced the proliferation of sarcoma cells in vitro in a dose-dependent manner (Fig. 8A&B). Moreover, wound healing assays indicated that ATBC stimulated the migration of both SW872 and MG-63 cells, with more pronounced effects at higher concentrations (Fig. 8C–F). These results suggest that ATBC enhances the proliferation and migration of sarcoma cells in vitro.

### ATBC regulates the mRNA expression of core sarcoma-related genes

**Fig. 9:**
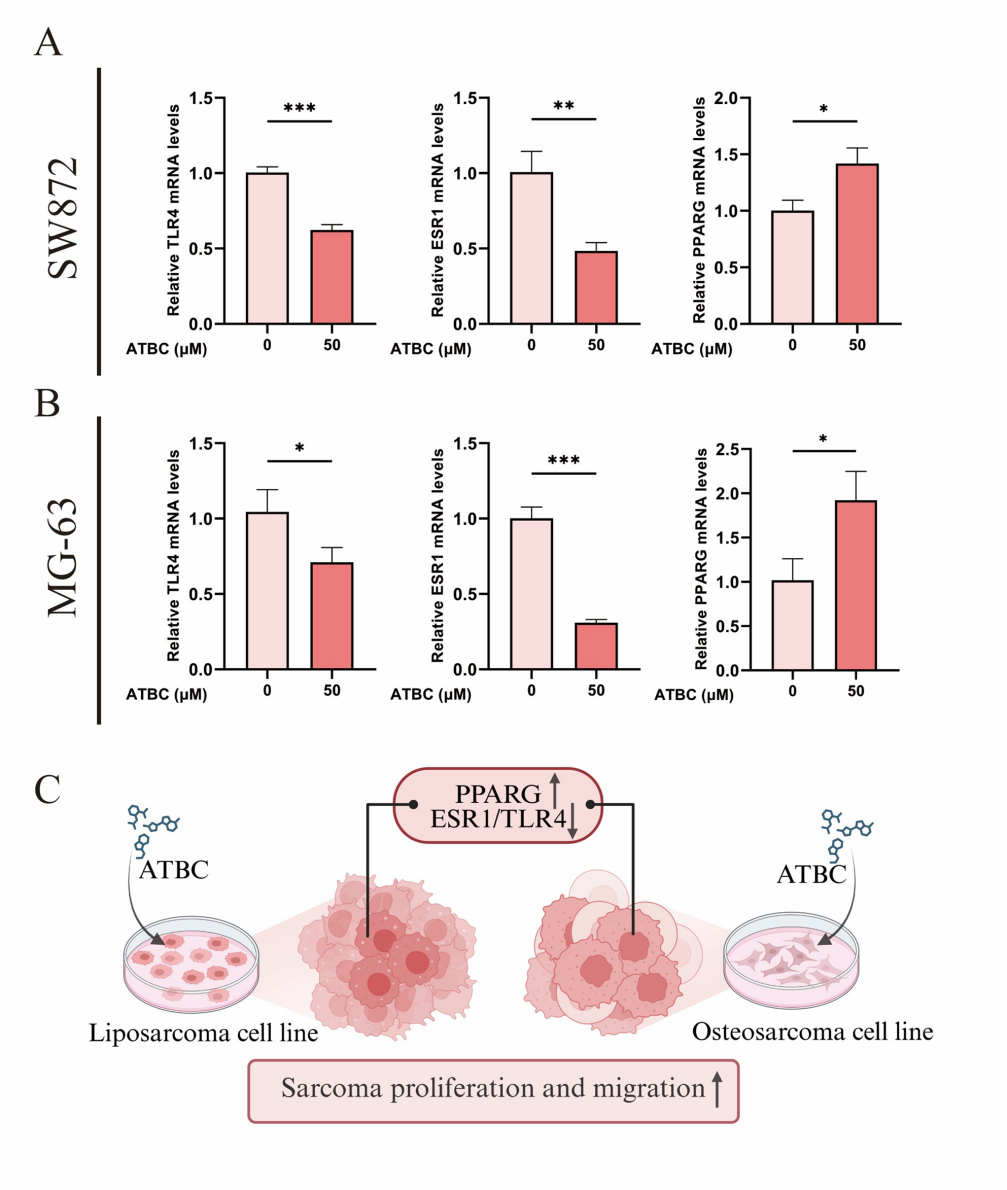
RT-qPCR analysis of representative hub genes in sarcoma cells following treatment with 50 µM ATBC. TLR4, ESR1, and PPARG mRNA expression in (**A**) SW872 and (**B**) MG-63 cells. Statistical evaluations were performed using two-tailed Student’s t-tests between the control and 50 µM ATBC groups. The symbols ns, *, **, and *** denote non-significance and significance levels at *P* < 0.05, *P* < 0.01, and *P* < 0.001, respectively

To further explore the transcriptional changes associated with ATBC exposure, the mRNA expression levels of selected core target genes were assessed by RT-qPCR. The results showed that 50 µM ATBC treatment decreased the mRNA expression of TLR4 and ESR1 in both SW872 and MG-63 cells, while increasing PPARG mRNA expression (Fig. 9A&B). These changes were directionally consistent with the survival analysis results and may provide preliminary support for the potential involvement of these genes in the biological effects of ATBC in sarcoma cells.

Additionally, although the initial Kaplan-Meier analysis did not reveal statistically significant associations between the expression levels of SIRT1, NFKB1, or PTGS2 and patient survival, ATBC treatment induced changes in their mRNA levels that showed a concordant trend with clinical prognosis (Figure S1). This observation requires further validation at the protein and pathway levels.

## Discussion

This research combines molecular docking, survival analysis, network toxicology, and in vitro experiments to investigate the potential role and underlying mechanisms of ATBC in the development and progression of sarcoma. As a widely used non-phthalate plasticizer, ATBC has been frequently detected in water bodies, indoor dust, and food packaging due to its high mobility and environmental persistence, making it an environmental contaminant of increasing concern [6, 29]. Although traditionally regarded as a relatively safe alternative, recent studies have revealed that ATBC exhibits endocrine-disrupting effects, which can lead to metabolic disorders and developmental toxicity in animals [9–11]. Environmental pollutants, particularly those with endocrine-disrupting properties, have been implicated in an elevated risk of tumorigenesis by multiple studies. For instance, plastic additives (such as DEP, DMP, and DOP) and PET microplastics have been shown to interfere with key signaling proteins such as MAPK1 and PIK3CA, thereby disrupting core pathways like PI3K-Akt-mTOR and facilitating the progression of multiple cancer types, such as breast cancer [30, 31]. However, the role and molecular mechanisms of ATBC in sarcoma remain unclear. Our research addresses this knowledge gap by suggesting, for the first time, that ATBC may exert tumor-promoting effects in sarcoma through modulation of multiple core targets, with several signaling pathways identified as candidate mechanisms requiring further validation.

Our study suggested that ATBC may interact with six core targets—TLR4, ESR1, PPARG, SIRT1, NFKB1, and PTGS2. Enrichment analyses indicated that these targets were associated with multiple oncogenic signaling pathways, including PI3K-Akt, HIF-1, AGE-RAGE, and sphingolipid signaling, which may be relevant to the observed promotion of sarcoma cell proliferation and migration in vitro. Through multi-database screening and protein-protein interaction network analysis, we first identified the aforementioned six core targets, which play critical roles in cell proliferation, apoptosis, immune response, and metabolic regulation. Molecular docking results suggested that ATBC could interact with all six core targets, with predicted binding energies ranging from -1.15 to -2.99 kcal/mol. Although these values indicate relatively weak interactions, they provide a preliminary theoretical basis for the hypothesis that ATBC may directly engage these proteins.

Functional analysis of the core targets revealed that ATBC may exert its tumor-promoting effects by regulating a complex molecular network. Among these, TLR4 and ESR1 were identified as potential tumor suppressors. As a key receptor in innate immunity, the activation of TLR4 is essential for the priming and activation of T cells [32, 33]. In osteosarcoma, stimulation of the TLR4 pathway has been shown to inhibit tumor progression through the activation of CD8 + T cells [34]. In this study, ATBC treatment significantly downregulated TLR4 expression, which is consistent with Kaplan–Meier analysis showing that low TLR4 expression is associated with poorer patient prognosis. This suggests that ATBC may create a favorable environment for tumor development by impairing TLR4-mediated anti-tumor immune surveillance. Similarly, ESR1 (estrogen receptor alpha) has been reported to suppress tumorigenesis in liver and breast cancer cells via regulation of CCN5 [35]. Our results demonstrated that ATBC downregulates ESR1 expression, and low ESR1 levels were correlated with adverse clinical outcomes, further supporting the hypothesis that ESR1 may act as a tumor suppressor in sarcoma.

In contrast, PPARG was identified in this study as a key pro-tumorigenic driver. Our survival analysis revealed that high PPARG expression significantly correlated with poorer sarcoma patient outcomes. This finding is highly consistent with previous studies: PPARG has been shown to promote angiogenesis by upregulating VEGF in cancers such as breast and prostate cancer [36, 37]. Moreover, recent research has also clearly demonstrated that it accelerates disease progression in osteosarcoma by regulating osteoclast proliferation [38]. In the present study, in vitro experiments confirmed that ATBC exposure significantly upregulates PPARG expression, while simultaneously enhancing the proliferation and migration abilities of sarcoma cells. Taken together, these findings support the potential involvement of PPARG in the effects of ATBC on sarcoma at both the molecular and cellular levels.

Other core targets, including SIRT1, NFKB1, and PTGS2, although not demonstrating significant prognostic value in our survival analysis, play important roles in tumor biology and are implicated in the potential carcinogenic mechanisms of ATBC. SIRT1, an NAD⁺-dependent deacetylase, participates in stress response and regulation of cell survival by deacetylating key proteins such as p53 and FOXO [39, 40]. In soft tissue sarcoma, high expression of SIRT1 is correlated with unfavorable clinical outcomes [41], and inhibition of SIRT1 has been shown to effectively suppress growth and induce cell death in pediatric soft tissue sarcoma [42]. In the present study, ATBC treatment resulted in a significant increase in SIRT1 expression in cells, suggesting that SIRT1 may also contribute to the pro-tumorigenic effects associated with ATBC exposure. NFKB1, a core component of the NF-κB signaling pathway, has been reported under certain contexts to exert anti-inflammatory and tumor-suppressive effects [43, 44]. Our experiments observed that ATBC downregulated NFKB1 expression, which may impair its potential tumor-suppressive function. Furthermore, PTGS2, a key enzyme involved in inflammation and shaping the tumor microenvironment, catalyzes the production of PGE₂, which can induce immunosuppression and promote tumor immune escape [45, 46]. In this study, ATBC downregulated PTGS2 expression, indicating its possible involvement in toxic effects via modulation of inflammatory signaling and the tumor microenvironment. This possibility is supported by recent evidence showing that alterations in the tumor immune microenvironment can critically influence tumor progression, immune cell infiltration, and therapeutic response [47–49]. Taken together, these findings suggest multiple hub genes. In particular, altered TLR4, NFKB1, and PTGS2 expression may contribute to changes in inflammatory signaling and tumor microenvironment regulation [34, 44–46], whereas dysregulation of ESR1, PPARG, and SIRT1 may further support a proliferative and stress-adaptive state [35, 38, 41]. These effects may converge on PI3K-Akt and HIF-1 signaling, thereby linking immune dysregulation, hypoxic adaptation, and tumor progression [50, 51]. From this perspective, ATBC exposure may contribute to sarcoma development through multi-target and microenvironment-related mechanisms, although the causal relationships still require further validation.

At the pathway level, KEGG enrichment analysis indicated that ATBC-related core targets were enriched in multiple cancer-associated pathways, providing a bioinformatic framework for interpreting its potential effects in sarcoma. Among these, aberrant activation of the PI3K-Akt pathway is highly prevalent in sarcomas and is linked to cell proliferation, survival, and chemotherapy resistance [50, 52]. Such activation is often linked to the inactivation of tumor suppressor genes such as PTEN [53–55]. Our enrichment results suggest that ATBC may interfere with the regulation of the PI3K-Akt pathway, although direct pathway activation remains to be experimentally validated. The HIF-1 signaling pathway plays a central role in the tumor hypoxic response, and its activation can promote angiogenesis [56–58]. In osteosarcoma, it drives cancer progression via activation of the AKT/Cyclin D1 axis [51]. Therefore, ATBC exposure may mimic or exacerbate hypoxic stress within tumors. Additionally, the sphingolipid signaling pathway warrants attention, as its key component SPK1 is critical for maintaining sphingolipid metabolic homeostasis and promoting cell survival [59]. Studies have confirmed that SPK1 is upregulated in osteosarcoma and contributes to doxorubicin resistance [60], suggesting a potential link between ATBC and sphingolipid signaling that warrants further investigation.

In terms of experimental validation, ATBC significantly enhanced the proliferation and migration capabilities of SW872 and MG-63 cells in vitro. In addition, RT-qPCR performed at the selected 50 µM concentration showed that ATBC treatment downregulated the expression of TLR4 and ESR1, while upregulating PPARG expression, which was consistent with the bioinformatic prediction at the target-gene level and with the observed clinical prognostic trends. Although the mRNA changes of SIRT1, NFKB1, and PTGS2 did not reach statistical significance in the survival analysis, their expression trends aligned with the direction of clinical outcomes, suggesting that they may play auxiliary roles in the ATBC toxicity network. Their specific mechanisms of action require further elucidation in more complex in vivo models. Overall, these experimental data support the involvement of selected core targets, but do not directly validate activation of the predicted signaling pathways.

Nevertheless, this research has a number of limitations. First, the cell line models used cannot fully recapitulate the complex heterogeneity of sarcomas in vivo or the tumor microenvironment, which includes immune cells and stromal components. Second, short-term drug interventions in vitro may not adequately reflect the pathophysiological processes associated with chronic, low-dose exposure to ATBC in humans. Accordingly, future studies should incorporate lower, more environmentally relevant exposure concentrations and, where possible, compare the in vitro concentrations used in the present study with available human exposure or tissue concentration data to better evaluate the real-world relevance of these findings. In addition, the survival analysis in this study was exploratory in nature, was based on a pooled public sarcoma cohort, and did not include multivariate adjustment or subtype-stratified analysis. Moreover, although RT-qPCR revealed transcriptional changes in several candidate genes, the present study did not include protein-level confirmation by Western blot, nor did it directly verify pathway activation. Furthermore, because RT-qPCR validation was performed at a single selected concentration, the dose dependence of ATBC-induced transcriptional changes could not be evaluated. Therefore, the mechanistic interpretation should be considered preliminary. Taken together, further studies are imperative to validate the tumor-promoting effects of ATBC using animal models, clinical models, and even epidemiological investigations, as well as to elucidate its direct regulatory impact on the tumor immune microenvironment.

It is important to note that the binding energies predicted by molecular docking in this study ranged from -1.15 to -2.99 kcal/mol, which are relatively modest compared to typical drug–target interactions. However, several considerations support the biological relevance of these findings. First, molecular docking serves primarily as a tool to predict potential binding modes and interaction residues, and its calculated binding energies are semi-quantitative; the presence of negative values, regardless of magnitude, indicates thermodynamically feasible interactions. Second, ATBC is an environmental plasticizer rather than an optimized pharmaceutical agent, and its interactions with protein targets are expected to be relatively weak, consistent with the concept that environmental chemicals often exert biological effects through low-affinity, multi-target mechanisms. Third, from a network toxicology perspective, ATBC is predicted to interact with multiple core targets. Even if individual binding affinities are modest, their combined perturbation of the molecular network can lead to significant phenotypic consequences, as supported by our in vitro observations of altered gene expression, enhanced proliferation, and increased migration in sarcoma cells. Moreover, the binding affinities predicted by molecular docking were relatively modest, and no positive control compounds were included for comparison. Future studies incorporating compounds with known binding affinities for these targets would help benchmark the docking results and more accurately assess the interaction strength between ATBC and its putative targets.

In summary, this study provides integrated evidence that ATBC may promote malignant phenotypes in sarcoma cells and may interact with several core targets, including TLR4, ESR1, PPARG, SIRT1, NFKB1, and PTGS2. Bioinformatic analyses further highlighted PI3K-Akt, HIF-1, and sphingolipid signaling as candidate pathways potentially involved in these effects, although direct mechanistic validation is still required. These findings not only provide a novel theoretical foundation for understanding the toxicological mechanisms of ATBC, but also suggest potential molecular targets for assessing its environmental health risks and developing preventive and therapeutic strategies for sarcoma.

## Electronic supplementary material

Below is the link to the electronic supplementary material.

Supplementary Material 1

## Data Availability

Data will be made available on request.

## References

[1] Testai E, Hartemann P, Rastogi SC, Bernauer U, Piersma A, De Jong W, et al. The safety of medical devices containing DEHP plasticized PVC or other plasticizers on neonates and other groups possibly at risk (2015 update). Regul Toxicol Pharmacol. 2016;76:209–10. 26854686 10.1016/j.yrtph.2016.01.013

[2] Hyeon K, Min Sun C, Young Seok J et al. In Sook K, Gi Beom K, In Yong B,. Pharmacokinetic properties of acetyl tributyl citrate, a pharmaceutical excipient. Pharmaceutics. 2018;10(4). 10.3390/pharmaceutics10040177 PMC6320780 30297635

[3] Lindsay MR, Nivedita S, Xiaosong L, Zelieann RC. Effects of oral exposure to the phthalate substitute acetyl tributyl citrate on female reproduction in mice. J Appl Toxicol. 2016;37(6). 10.1002/jat.3413 PMC5400675 27866379

[4] Akira T, Junko I-M, Kazusa N, Hideyo T, Yasuhiro T, Noriyuki K. Acetyl tributyl citrate, the most widely used phthalate substitute plasticizer, induces cytochrome p450 3a through steroid and xenobiotic receptor. Toxicol Sci. 2011;123(2). 10.1093/toxsci/kfr178 21742782

[5] Paul JL, Russ H, Chris G, Holger MK, Philip EM, Bernard AS, et al. Assessment of phthalates/phthalate alternatives in children’s toys and childcare articles: Review of the report including conclusions and recommendation of the Chronic Hazard Advisory Panel of the Consumer Product Safety Commission. J Expo Sci Environ Epidemiol. 2015;25(4). 10.1038/jes.2015.33 25944701

[6] Lin X, Lin K, Lai Y, Peng Q, Xu M, Xu Y, et al. Effect of Acetyl tributyl citrate on bone metabolism based on network toxicology and molecular docking technology. Ecotoxicol Environ Saf. 2025;289:117434. 39615059 10.1016/j.ecoenv.2024.117434

[7] Takeshita A, Igarashi-Migitaka J, Nishiyama K, Takahashi H, Takeuchi Y, Koibuchi N. Acetyl tributyl citrate, the most widely used phthalate substitute plasticizer, induces cytochrome p450 3a through steroid and xenobiotic receptor. Toxicol Sci. 2011;123(2):460–70. 21742782 10.1093/toxsci/kfr178

[8] Alves A, Giovanoulis G, Nilsson U, Erratico C, Lucattini L, Haug LS, et al. Case Study on Screening Emerging Pollutants in Urine and Nails. Environ Sci Technol. 2017;51(7):4046–53. 28293951 10.1021/acs.est.6b05661

[9] Rasmussen LM, Sen N, Liu X, Craig ZR. Effects of oral exposure to the phthalate substitute acetyl tributyl citrate on female reproduction in mice. J Appl Toxicol. 2017;37(6):668–75. 27866379 10.1002/jat.3413 PMC5400675

[10] Zhang Y, Zhang Z, Zhu S, Liu L, Liu X, Yang X. Acetyl tributyl citrate exposure at seemingly safe concentrations induces adverse effects in different genders of type 2 diabetes mice, especially brain tissue. Toxics. 2023;11(10). 10.3390/toxics11100877 PMC10610634 37888727

[11] Horie Y, Yap CK, Okamura H. Developmental toxicity and thyroid hormone-disrupting effects of acetyl tributyl citrate in zebrafish and Japanese medaka. J Hazard Mater Adv. 2022;8.

[12] Guo DQ, Fang YM, Sun ZD, Zeng YF, Wang GD, Liang JW. Analyzing the potential targets and mechanisms of liver damage induced by acetyl tributyl citrate plasticizer using network toxicology, molecular docking and in vitro experiments. Front Pharmacol. 2025;16:1636576. 40741007 10.3389/fphar.2025.1636576 PMC12307183

[13] Zhang D, Zhang W, Liu H, Huang S, Huang W, Zhu Y, et al. Intergenerational metabolism-disrupting effects of maternal exposure to plasticizer acetyl tributyl citrate (ATBC). Environ Int. 2024;191:108967. 39217724 10.1016/j.envint.2024.108967

[14] Murphey MD, Kransdorf MJ. Staging and Classification of Primary Musculoskeletal Bone and Soft-Tissue Tumors According to the 2020 WHO Update, From the AJR Special Series on Cancer Staging. AJR Am J Roentgenol. 2021;217(5):1038–52. 33852362 10.2214/AJR.21.25658

[15] Ozaki T, Flege S, Liljenqvist U, Hillmann A, Delling G, Salzer-Kuntschik M, et al. Osteosarcoma of the spine: experience of the Cooperative Osteosarcoma Study Group. Cancer. 2002;94(4):1069–77. 11920477

[16] Kansara M, Teng MW, Smyth MJ, Thomas DM. Translational biology of osteosarcoma. Nat Rev Cancer. 2014;14(11):722–35. 25319867 10.1038/nrc3838

[17] Liu W, Cheng H, Huang Z, Li Y, Zhang Y, Yang Y, et al. The correlation between clinical outcomes and genomic analysis with high risk factors for the progression of osteosarcoma. Mol Oncol. 2024;18(4):939–55. 37727135 10.1002/1878-0261.13526 PMC10994228

[18] Cancer Genome Atlas Research Network. Comprehensive and Integrated Genomic Characterization of Adult Soft Tissue Sarcomas. Cell. 2017;171(4):950–e6528. 29100075 10.1016/j.cell.2017.10.014 PMC5693358

[19] Wang X, Ye J, Liu D, Yan L, Li S, Lv A, et al. Bibliometric analysis of liposarcomas treatment from 2004 to 2023. J Cancer Res Clin Oncol. 2025;151(2):47. 39856436 10.1007/s00432-025-06094-0 PMC11762222

[20] Ritter J, Bielack SS, Osteosarcoma. Ann Oncol. 2010;21(Suppl 7):vii320–5. 20943636 10.1093/annonc/mdq276

[21] Leite TC, Watters RJ, Weiss KR, Intini G. Avenues of research in dietary interventions to target tumor metabolism in osteosarcoma. J Transl Med. 2021;19(1):450. 34715874 10.1186/s12967-021-03122-8 PMC8555297

[22] Rickel K, Fang F, Tao J. Molecular genetics of osteosarcoma. Bone. 2017;102:69–79. 27760307 10.1016/j.bone.2016.10.017 PMC5393957

[23] Edwards D, Voronina A, Attwood K, Grand’Maison A. Association between occupational exposures and sarcoma incidence and mortality: systematic review and meta-analysis. Syst Rev. 2021;10(1):231. 34389054 10.1186/s13643-021-01769-4 PMC8364027

[24] Hopkins AL. Network pharmacology. Nat Biotechnol. 2007;25(10):1110–1. 17921993 10.1038/nbt1007-1110

[25] Hopkins AL. Network pharmacology: the next paradigm in drug discovery. Nat Chem Biol. 2008;4(11):682–90. 18936753 10.1038/nchembio.118

[26] Del Giudice G, Serra A, Pavel A, Torres Maia M, Saarimäki LA, Fratello M, et al. A Network Toxicology Approach for Mechanistic Modelling of Nanomaterial Hazard and Adverse Outcomes. Adv Sci (Weinh). 2024;11(32):e2400389. 38923832 10.1002/advs.202400389 PMC11348149

[27] He J, Zhu X, Xu K, Li Y, Zhou J. Network toxicological and molecular docking to investigate the mechanisms of toxicity of agricultural chemical Thiabendazole. Chemosphere. 2024;363:142711. 38964723 10.1016/j.chemosphere.2024.142711

[28] Li T, Guo R, Zong Q, Ling G. Application of molecular docking in elaborating molecular mechanisms and interactions of supramolecular cyclodextrin. Carbohydr Polym. 2022;276:118644. 34823758 10.1016/j.carbpol.2021.118644

[29] Tao L, Tan H, Qiao X, Li L, Yu Y, Xie J, et al. Emerging Plasticizers in South China House Dust and Hand Wipes: Calling for Potential Concern? Environ Sci Technol. 2022;56(17):12190–9. 35975842 10.1021/acs.est.2c02106

[30] He N, Zhang J, Liu M, Yin L. Elucidating the mechanism of plasticizers inducing breast cancer through network toxicology and molecular docking analysis. Ecotoxicol Environ Saf. 2024;284:116866. 39178760 10.1016/j.ecoenv.2024.116866

[31] Yang K, Zhou X, Wu K, Wu J, Huang C, Yang L. Multi-omics reveals the polyethylene terephthalate carcinogenicity: Cancer progression and immune microenvironment. Ecotoxicol Environ Saf. 2025;302:118522. 40532600 10.1016/j.ecoenv.2025.118522

[32] Bock M, Bergmann CB, Jung S, Kalbitz M, Relja B, Huber-Wagner S, et al. The posttraumatic activation of CD4 + T regulatory cells is modulated by TNFR2- and TLR4-dependent pathways, but not by IL-10. Cell Immunol. 2018;331:137–45. 29954581 10.1016/j.cellimm.2018.06.009

[33] Dias ASO, Sacramento PM, Lopes LM, Sales MC, Castro C, Araújo A, et al. TLR-2 and TLR-4 agonists favor expansion of CD4(+) T cell subsets implicated in the severity of neuromyelitis optica spectrum disorders. Mult Scler Relat Disord. 2019;34:66–76. 31229737 10.1016/j.msard.2019.06.018

[34] Yahiro K, Matsumoto Y, Yamada H, Endo M, Setsu N, Fujiwara T, et al. Activation of TLR4 signaling inhibits progression of osteosarcoma by stimulating CD8-positive cytotoxic lymphocytes. Cancer Immunol Immunother. 2020;69(5):745–58. 32047957 10.1007/s00262-020-02508-9 PMC11027819

[35] Zheng D, Bashir M, Li Z. ERα prevents tumorigenesis of both liver and breast cancer cells through CCN5. Biochem Biophys Res Commun. 2023;672:103–12. 37343316 10.1016/j.bbrc.2023.06.018

[36] Yun SH, Han SH, Park JI. Peroxisome Proliferator-Activated Receptor γ and PGC-1α in Cancer: Dual Actions as Tumor Promoter and Suppressor. PPAR Res. 2018;2018:6727421. 29599799 10.1155/2018/6727421 PMC5828371

[37] Forootan FS, Forootan SS, Gou X, Yang J, Liu B, Chen D, et al. Fatty acid activated PPARγ promotes tumorigenicity of prostate cancer cells by up regulating VEGF via PPAR responsive elements of the promoter. Oncotarget. 2016;7(8):9322–39. 26814431 10.18632/oncotarget.6975 PMC4891043

[38] Sun L, Zhang J, Xiahou Z, Zhao Z, Liang Y. Single-cell RNA sequencing revealed PPARG promoted osteosarcoma progression: based on osteoclast proliferation. Front Immunol. 2024;15:1506225. 39936154 10.3389/fimmu.2024.1506225 PMC11810940

[39] Chen WY, Wang DH, Yen RC, Luo J, Gu W, Baylin SB. Tumor suppressor HIC1 directly regulates SIRT1 to modulate p53-dependent DNA-damage responses. Cell. 2005;123(3):437–48. 16269335 10.1016/j.cell.2005.08.011

[40] Brunet A, Sweeney LB, Sturgill JF, Chua KF, Greer PL, Lin Y, et al. Stress-dependent regulation of FOXO transcription factors by the SIRT1 deacetylase. Science. 2004;303(5666):2011–5. 14976264 10.1126/science.1094637

[41] Kim JR, Moon YJ, Kwon KS, Bae JS, Wagle S, Yu TK, et al. Expression of SIRT1 and DBC1 is associated with poor prognosis of soft tissue sarcomas. PLoS ONE. 2013;8(9):e74738. 24019980 10.1371/journal.pone.0074738 PMC3760851

[42] Ma L, Maruwge W, Strambi A, D’Arcy P, Pellegrini P, Kis L, et al. SIRT1 and SIRT2 inhibition impairs pediatric soft tissue sarcoma growth. Cell Death Dis. 2014;5(10):e1483. 25341037 10.1038/cddis.2014.385 PMC4237232

[43] Aggarwal BB. Nuclear factor-kappaB: the enemy within. Cancer Cell. 2004;6(3):203–8. 15380510 10.1016/j.ccr.2004.09.003

[44] Cartwright T, Perkins ND, C LW. NFKB1: a suppressor of inflammation, ageing and cancer. Febs j. 2016;283(10):1812–22. 26663363 10.1111/febs.13627

[45] Obermajer N, Muthuswamy R, Lesnock J, Edwards RP, Kalinski P. Positive feedback between PGE2 and COX2 redirects the differentiation of human dendritic cells toward stable myeloid-derived suppressor cells. Blood. 2011;118(20):5498–505. 21972293 10.1182/blood-2011-07-365825 PMC3217352

[46] Kalinski P. Regulation of immune responses by prostaglandin E2. J Immunol. 2012;188(1):21–8. 22187483 10.4049/jimmunol.1101029 PMC3249979

[47] Xia W, Liu J, Chen R, Feng J, Wu L, Wang Y, et al. Molecular subtypes and prognostic signature rooted in disulfidptosis highlight tumor microenvironment in lung adenocarcinoma. Chin J Cancer Res. 2025;37(5):796–820. 41246257 10.21147/j.issn.1000-9604.2025.05.11 PMC12614471

[48] Gong Z, Du M, Li Y, Ye B, Huang Y, Gong H, et al. Machine learning identifies TIME subtypes linking EGFR mutations and immune states in lung adenocarcinoma. NPJ Digit Med. 2025;8(1):796. 41299024 10.1038/s41746-025-02172-2 PMC12749019

[49] Zhang P, Liang X, Ye B, Wang X, Wang Y, Gong Z, et al. Metabolic reprogramming signature predicts immunotherapy efficacy in lung adenocarcinoma: Targeting SLC25A1 to overcome immune resistance. Chin J Cancer Res. 2025;37(6):1000–19. 41536497 10.21147/j.issn.1000-9604.2025.06.11 PMC12796613

[50] Li H, Shen X, Ma M, Liu W, Yang W, Wang P, et al. ZIP10 drives osteosarcoma proliferation and chemoresistance through ITGA10-mediated activation of the PI3K/AKT pathway. J Exp Clin Cancer Res. 2021;40(1):340. 34706747 10.1186/s13046-021-02146-8 PMC8549349

[51] Zhang B, Li YL, Zhao JL, Zhen O, Yu C, Yang BH, et al. Hypoxia-inducible factor-1 promotes cancer progression through activating AKT/Cyclin D1 signaling pathway in osteosarcoma. Biomed Pharmacother. 2018;105:1–9. 29807229 10.1016/j.biopha.2018.03.165

[52] Li A, Wang S, Nie J, Xiao S, Xie X, Zhang Y, et al. USP3 promotes osteosarcoma progression via deubiquitinating EPHA2 and activating the PI3K/AKT signaling pathway. Cell Death Dis. 2024;15(3):235. 38531846 10.1038/s41419-024-06624-7 PMC10965993

[53] Yu C, Zhang B, Li YL, Yu XR. SIX1 reduces the expression of PTEN via activating PI3K/AKT signal to promote cell proliferation and tumorigenesis in osteosarcoma. Biomed Pharmacother. 2018;105:10–7. 29807230 10.1016/j.biopha.2018.04.028

[54] Li C, Xu B, Miu X, Deng Z, Liao H, Hao L. Inhibition of miRNA-21 attenuates the proliferation and metastasis of human osteosarcoma by upregulating PTEN. Exp Ther Med. 2018;15(1):1036–40. 29434694 10.3892/etm.2017.5477 PMC5772948

[55] Zhu Y, Tang H, Zhang L, Gong L, Wu G, Ni J, et al. Suppression of miR-21-3p enhances TRAIL-mediated apoptosis in liver cancer stem cells by suppressing the PI3K/Akt/Bad cascade via regulating PTEN. Cancer Manag Res. 2019;11:955–68. 30774424 10.2147/CMAR.S183328 PMC6349085

[56] Wang GL, Jiang BH, Rue EA, Semenza GL. Hypoxia-inducible factor 1 is a basic-helix-loop-helix-PAS heterodimer regulated by cellular O2 tension. Proc Natl Acad Sci U S A. 1995;92(12):5510–4. 7539918 10.1073/pnas.92.12.5510 PMC41725

[57] Basheeruddin M, Qausain S. Hypoxia-Inducible Factor 1-Alpha (HIF-1α) and Cancer: Mechanisms of Tumor Hypoxia and Therapeutic Targeting. Cureus. 2024;16(10):e70700. 39493156 10.7759/cureus.70700 PMC11529905

[58] Magar AG, Morya VK, Kwak MK, Oh JU, Noh KC. A molecular perspective on HIF-1α and angiogenic stimulator networks and their role in solid tumors: an update. Int J Mol Sci. 2024;25(6). 10.3390/ijms25063313 PMC10970012 38542288

[59] Kunkel GT, Maceyka M, Milstien S, Spiegel S. Targeting the sphingosine-1-phosphate axis in cancer, inflammation and beyond. Nat Rev Drug Discov. 2013;12(9):688–702. 23954895 10.1038/nrd4099 PMC3908769

[60] Ren X, Su C. Sphingosine kinase 1 contributes to doxorubicin resistance and glycolysis in osteosarcoma. Mol Med Rep. 2020;22(3):2183–90. 32705189 10.3892/mmr.2020.11295 PMC7411368

