# Supplementary material for: Integrative bioinformatics, network toxicology, and molecular docking elucidate molecular mechanisms of ATBC-induced sarcoma progression with experimental validation
Source: BMC Pharmacol Toxicol. 2026 Apr 24;27:85. doi: 10.1186/s40360-026-01141-z (PMC13245103; doi:10.1186/s40360-026-01141-z)
Supplement: Supplementary file 1 — Supplementary Material 1 [file 40360_2026_1141_MOESM1_ESM.docx]

**Supplementary Data**

**Supplementary Figures**

**Figure. S1. RT-qPCR was performed to measure the mRNA expression of core targets**

**
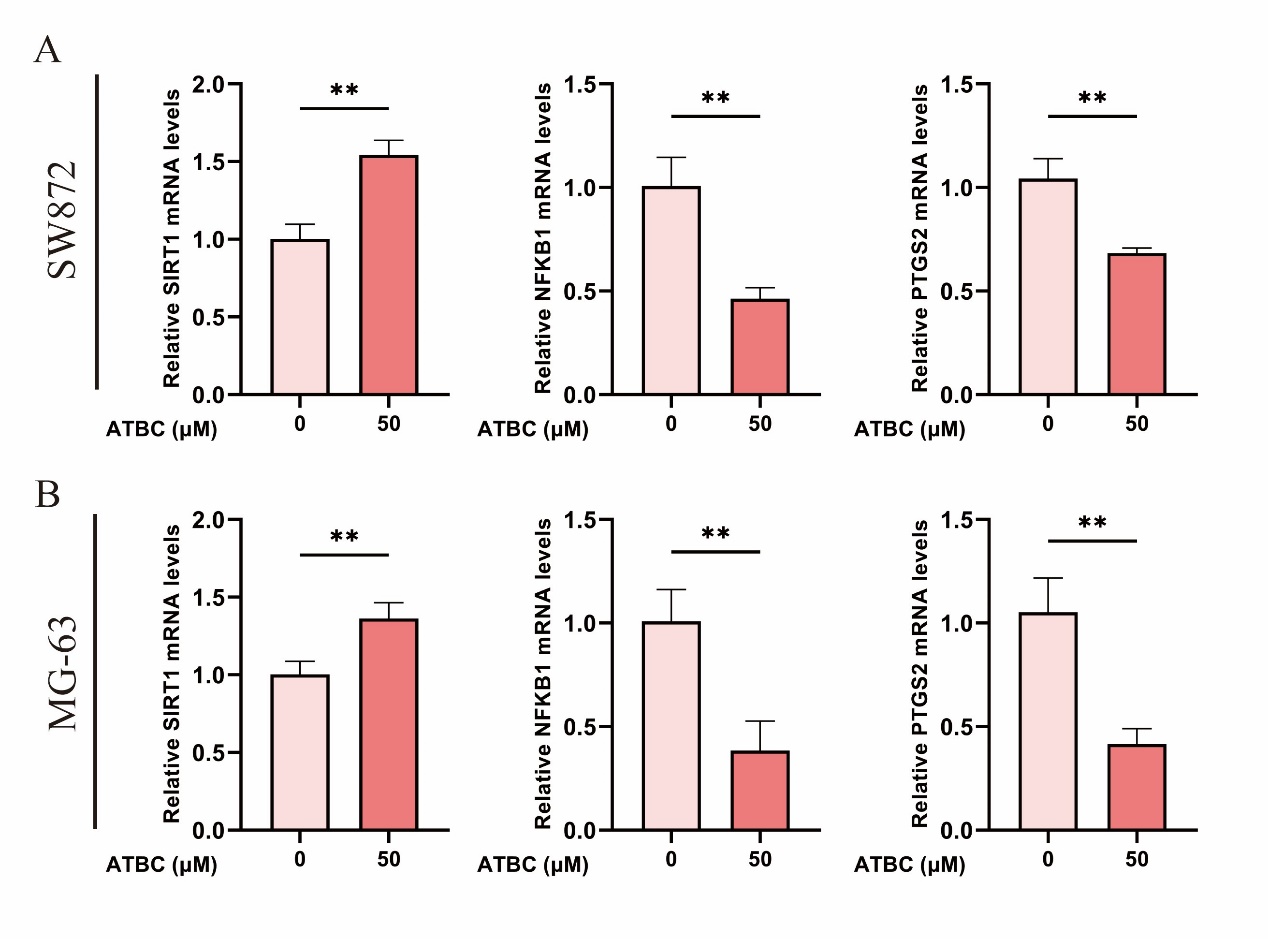
**

**Supplementary Figure. S1. RT-qPCR was performed to measure the mRNA expression of core targets.** SIRT1, NF-KB1, and PTGS2 mRNA expression in (A) SW872 and (B) MG-63 cells. Statistical evaluations were performed using two-tailed Student's t-tests. The symbols ns, *, **, and *** denote non-significance and significance levels at P < 0.05, P < 0.01, and P < 0.001, respectively.

**Figure. S2. Original wound-healing images corresponding to Figure 8C and Figure 8E**

**A. SW872 (corresponding to Figure 8C)**

| **Group** | **0 h** | **24h** | **48 h** |
| --- | --- | --- | --- |
| **Control** | 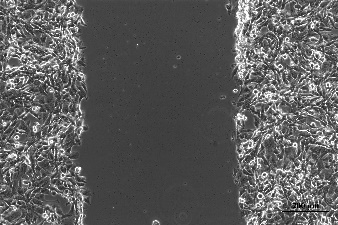 | 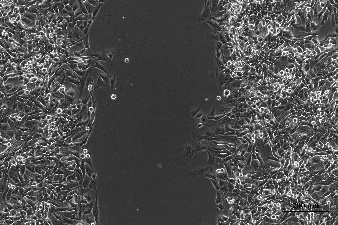 | 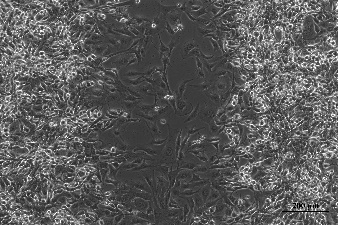 |
| **25 μM** | 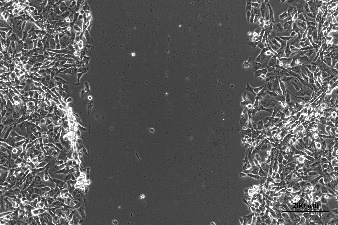 | 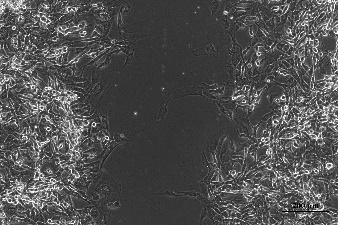 | 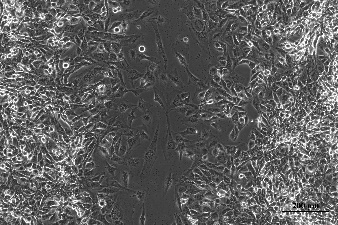 |
| **50 μM** | 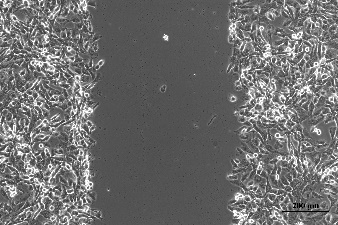 | 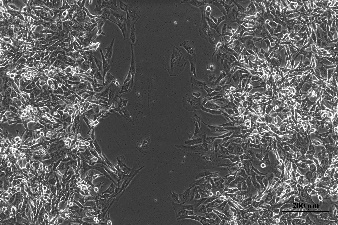 | 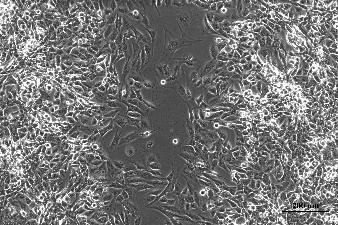 |

**B. MG-63 (corresponding to Figure 8E)**

| **Group** | **0 h** | **36 h** | **48 h** |
| --- | --- | --- | --- |
| **Control** | 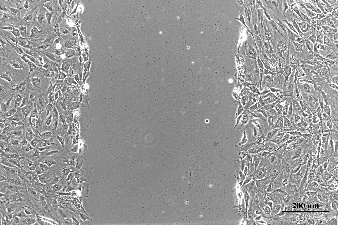 | 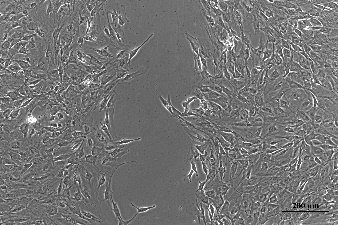 | 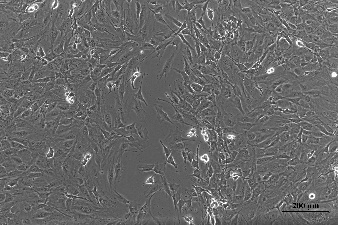 |
| **25 μM** | 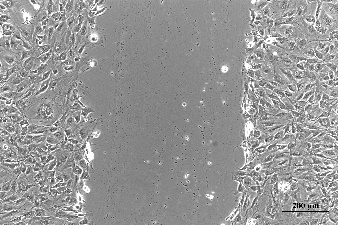 | 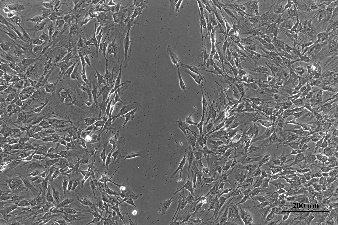 | 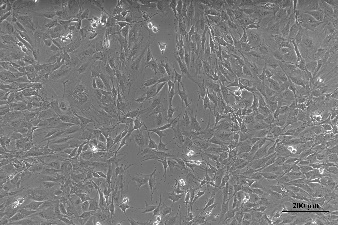 |
| **50 μM** | 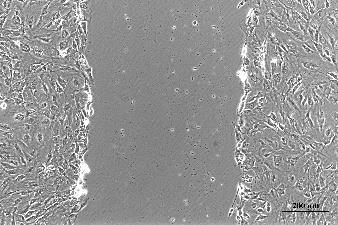 | 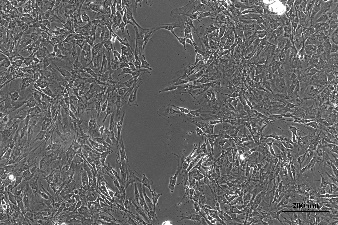 | 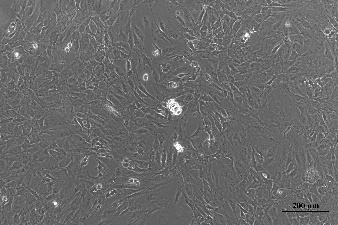 |

**Supplementary Figure. S2. Original wound-healing images corresponding to Figure 8C and Figure 8E.** Original wound-healing images of SW872 and MG-63 cells corresponding to the representative images shown in Figure 8C and Figure 8E. Images were acquired at 0, 24 (36), and 48 h after treatment with 0, 25, or 50μM ATBC.

**Figure. S3. Representative melt-curve analysis of RT-qPCR products**

| 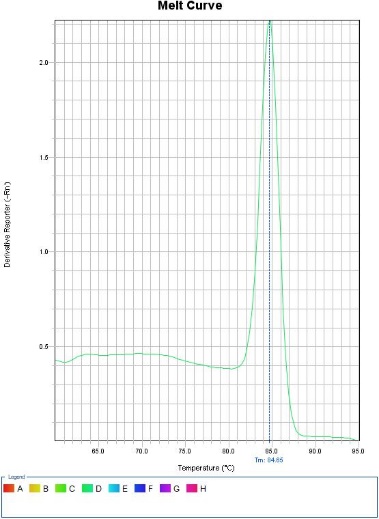 | **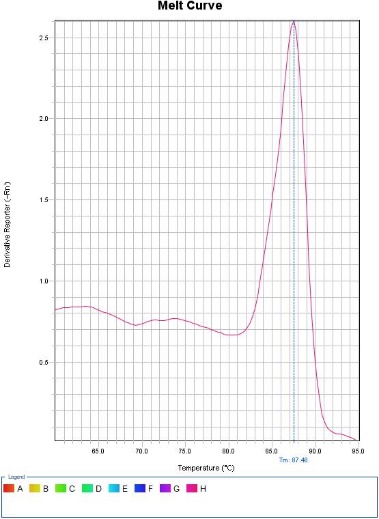** | **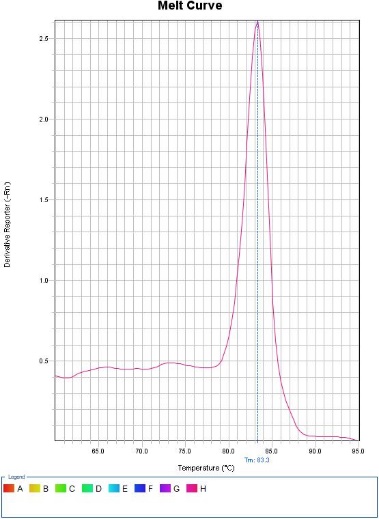** |
| --- | --- | --- |
| (A) TLR4 | (B) ESR1 | (C) PPARG |
| 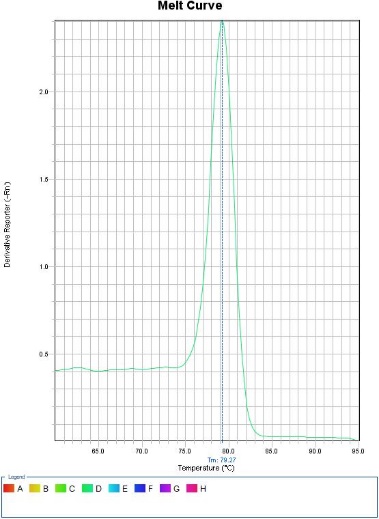 | 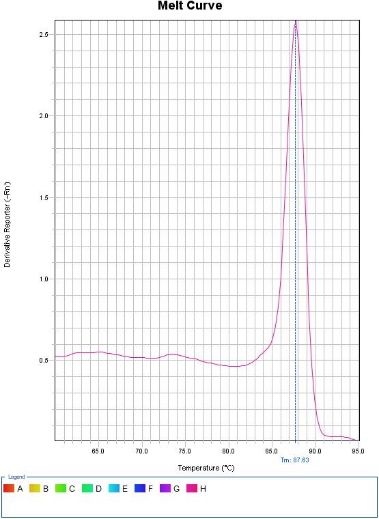 | 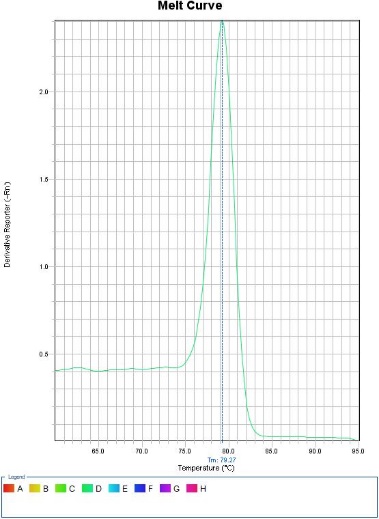 |
| (D) SIRT1 | (E) NFKB1 | (F) PTGS2 |

**Supplementary Figure. S3. Representative melt-curve analysis of RT-qPCR products.** Representative melt curves for TLR4, ESR1, PPARG, SIRT1, NFKB1, and PTGS2 in SW872 cells are shown. A single peak was observed for each primer pair, supporting the specificity of amplification.

**Supplementary Table**

**Table. S1. Normalized relative mRNA expression data from RT-qPCR analysis.**

| **Cell line** | **Gene** | **Group** | **Replicate 1** | **Replicate 2** | **Replicate 3** | **Mean ± SD** |  |
| --- | --- | --- | --- | --- | --- | --- | --- |
| SW872 | TLR4 | Control | 1.013957791 | 0.962465077 | 1.036566139 | 1.0043± 0.0380 |  |
| SW872 | TLR4 | 50 μM ATBC | 0.637226668 | 0.583312085 | 0.64946916 | 0.6233± 0.0352 |  |
| SW872 | ESR1 | Control | 0.874619301 | 0.994071399 | 1.150173519 | 1.0063± 0.1382 |  |
| SW872 | ESR1 | 50 μM ATBC | 0.427985898 | 0.537477264 | 0.486712762 | 0.4841 ± 0.0548 | |
| SW872 | PPARG | Control | 1.069808333 | 1.040890805 | 0.898025878 | 1.0029 ± 0.0920 | |
| SW872 | PPARG | 50 μM ATBC | 1.567298062 | 1.392141991 | 1.295783842 | 1.4184 ± 0.1377 | |
| SW872 | SIRT1 | Control | 0.895724368 | 1.038857492 | 1.074656413 | 1.0031 ± 0.0947 | |
| SW872 | SIRT1 | 50 μM ATBC | 1.487777619 | 1.486806262 | 1.65242548 | 1.5423 ± 0.0954 | |
| SW872 | NFKB1 | Control | 0.848312646 | 1.105002752 | 1.06679433 | 1.0067 ± 0.1385 | |
| SW872 | NFKB1 | 50 μM ATBC | 0.483791107 | 0.503683134 | 0.401164254 | 0.4629 ± 0.0544 | |
| SW872 | PTGS2 | Control | 1.130456247 | 1.058832389 | 0.939065972 | 1.0428 ± 0.0967 | |
| SW872 | PTGS2 | 50 μM ATBC | 0.69245609 | 0.701872991 | 0.655391894 | 0.6832 ± 0.0246 | |
| MG-63 | TLR4 | Control | 1.21227549 | 0.939872028 | 0.982167497 | 1.0448 ± 0.1466 | |
| MG-63 | TLR4 | 50 μM ATBC | 0.61509639 | 0.808963833 | 0.707886164 | 0.7106 ± 0.0970 | |
| MG-63 | ESR1 | Control | 1.084210625 | 0.98504475 | 0.936333101 | 1.0019 ± 0.0754 | |
| MG-63 | ESR1 | 50 μM ATBC | 0.313444322 | 0.286949788 | 0.328041958 | 0.3095 ± 0.0208 | |
| MG-63 | PPARG | Control | 0.928202013 | 0.833410953 | 1.292701611 | 1.0181 ± 0.2425 | |
| MG-63 | PPARG | 50 μM ATBC | 1.874033013 | 1.621925433 | 2.269460727 | 1.9218 ± 0.3264 | |
| MG-63 | SIRT1 | Control | 1.009988016 | 0.914424572 | 1.082769195 | 1.0024 ± 0.0844 | |
| MG-63 | SIRT1 | 50 μM ATBC | 1.471534945 | 1.34332873 | 1.271874663 | 1.3622 ± 0.1012 | |
| MG-63 | NFKB1 | Control | 0.843176317 | 1.032424131 | 1.148744523 | 1.0081 ± 0.1542 | |
| MG-63 | NFKB1 | 50 μM ATBC | 0.454190105 | 0.478192439 | 0.219333378 | 0.3839 ± 0.1430 | |
| MG-63 | PTGS2 | Control | 1.120072424 | 0.861504619 | 1.172414964 | 1.0513 ± 0.1665 | |
| MG-63 | PTGS2 | 50 μM ATBC | 0.359854755 | 0.383443293 | 0.501737793 | 0.4150 ± 0.0760 | |

**Supplementary Table. S1. Normalized relative mRNA expression data from RT-qPCR analysis.** Processed RT-qPCR results for target genes in SW872 and MG-63 cells following normalization to GAPDH. Relative expression values were calculated using the 2^−ΔΔCt method and are presented as normalized expression values for each biological replicate.
